# Physics-informed multi-task learning for permeability prediction and probabilistic HFU modeling: a case study from the Lower Bahariya Reservoir, Shahd SE field Egypt

**DOI:** 10.1038/s41598-026-61051-2

**Published:** 2026-07-19

**Authors:** Khaled Saleh, Walid M. Mabrouk, Ahmed M. Metwally

**Affiliations:** 1 Department of Geophysics, Faculty of Science, Cairo University, Giza, 12613 Egypt; 2 PetroShahd Company, Zahraa Maadi, Cairo, Egypt

**Keywords:** Permeability prediction, Hydraulic flow units (HFU), PINN, Multi-tasking PINN, Core analysis, Lower Bahariya reservoir, Engineering, Mathematics and computing, Solid Earth sciences

## Abstract

Accurate permeability prediction is essential for reliable reservoir characterization and simulation, yet remains challenging due to complex nonlinear relationships and subsurface heterogeneity. Conventional hydraulic flow unit (HFU) methods rely on discrete rock typing and fixed porosity–permeability relationships, limiting their ability to capture continuous variations. Physics-informed neural networks (PINNs) offer a data-driven alternative with embedded physical constraints, but their effectiveness is often limited by weak enforcement of physics during inference. In this study, a physics-guided multi-task neural network (MT-PINN) is proposed to simultaneously predict permeability and hydraulic flow units within a unified framework. The model integrates data-driven learning with physics-based relationships and probabilistic rock typing, enabling permeability to be estimated as a weighted combination of multiple flow units and allowing smoother transitions between facies. The proposed approach was evaluated using core and well log data and compared against conventional HFU and standard PINN methods. Within the studied dataset, the MT-PINN demonstrated improved predictive performance, with a higher correlation coefficient ($\:R\:=\:0.936$) compared to HFU ($\:R\:=\:0.89$) and PINN ($\:R\:=\:0.90$), along with a reduction in log-scale error. The model also provides more continuous and stable permeability predictions along depth. In addition to permeability estimation, the MT-PINN outputs both discrete HFU classifications and associated class probabilities, which can be incorporated into 3D stochastic reservoir modeling workflows for uncertainty-aware multi-realization analysis. The proposed framework demonstrates the potential to integrate traditional petrophysical methods with modern machine learning techniques, providing a promising workflow for permeability prediction and reservoir characterization within the studied reservoir.

## Introduction

Accurate permeability prediction is a fundamental component of reservoir characterization, directly influencing flow simulation, reserves estimation, and field development planning^1,2^. However, permeability is highly heterogeneous and controlled by complex interactions among pore structure, sedimentological facies, and diagenetic processes, making its estimation from well logs a challenging task^3^. Core measurements provide reliable permeability values but are sparse and expensive, motivating the development of predictive models that integrate well log data with petrophysical understanding^4,5^.

Traditional approaches to permeability prediction are largely based on hydraulic flow unit (HFU) concepts, where reservoir rocks are classified into units with similar pore throat characteristics and modeled using empirical relationships such as $\:k=a{{\upphi\:}}^{b}$. The seminal work of Amaefule et al. (1993) established the foundation for HFU-based reservoir characterization by linking permeability to porosity through flow zone indicators^6^. These methods are grounded in strong physical principles and have been widely applied in both clastic and carbonate reservoirs^7^. Nevertheless, HFU-based approaches rely on discrete rock typing, which introduces abrupt transitions and is sensitive to classification uncertainties. Furthermore, these methods simplify reservoir heterogeneity into homogeneous units and do not explicitly capture the complex nonlinear relationships between well logs and permeability, limiting their generalization capability in heterogeneous reservoirs^8,9^.

To overcome these limitations, machine learning (ML) techniques have been increasingly adopted to directly map well logs to permeability. Different machine learning studies explored support vector machines and ensemble learning methods for improved performance^10–13^. While applications demonstrated the effectiveness of artificial neural networks in predicting permeability from well logs^4,14,15,15–17,19,21^ and the ability of deep learning frameworks of capturing complex nonlinear relationships in subsurface data^22,23^. Despite their predictive capability, these methods are fundamentally data-driven and lack physical constraints, which can lead to non-physical predictions and poor extrapolation behavior outside the training domain.

Physics-informed neural networks (PINNs) have emerged as a promising framework for integrating physical knowledge into machine learning models by embedding governing equations into the loss function^24^. This approach enables the model to honor physical relationships while maintaining flexibility in learning from data. Recent developments in physics-informed machine learning have demonstrated its effectiveness across various scientific domains^25^. In permeability prediction, PINNs can incorporate petrophysical relationships, such as HFU-based equations, to constrain model outputs^26^. However, these methods still rely on explicit rock type labels, and thus inherit the limitations associated with discrete facies classification, including sensitivity to misclassification and inability to represent transitional geological behavior^8,9^.

Despite these advances, a fundamental gap remains: existing approaches do not simultaneously integrate rock typing, physics-based modeling, and data-driven learning in a continuous and uncertainty-aware framework. This limitation becomes particularly critical in heterogeneous reservoirs, where facies boundaries are inherently gradual rather than discrete.

To address this challenge, this study proposes a multi-task physics-guided neural network (MT-PINN Algorithm) framework that reformulates the classical HFU workflow into a probabilistic and differentiable representation. Instead of assigning a single rock type, the model predicts a distribution over possible flow units and computes permeability as a weighted combination of rock-type-dependent physical models. This formulation can be interpreted as conceptually analogous to mixture-of-experts models^27,28^, where each rock type acts as a local expert governed by a physics-based permeability–porosity relationship. By enabling soft rock typing, the proposed approach captures uncertainty in facies classification while preserving physically consistent relationships between porosity and permeability.

The main contributions of this work are as follows:

1. reformulating the classical HFU-based permeability model into a probabilistic framework using soft rock typing;
2. integrating physics-guided constraints with multi-task learning to jointly predict rock type and permeability;
3. developing a unified model that bridges the gap between discrete petrophysical methods and continuous machine learning approaches; and.
4. demonstrating improved robustness and generalization compared to HFU-based methods, conventional ML models, and standard PINN formulations.

Although the proposed framework shares certain conceptual similarities with probabilistic classification and mixture-of-experts approaches, its primary contribution lies in the integration of probabilistic HFU modeling, multi-task learning, and physics-guided petrophysical constraints within a unified reservoir characterization workflow. Unlike conventional mixture-of-experts formulations that rely primarily on data-driven expert combinations, the proposed framework incorporates physically interpretable HFU-dependent porosity–permeability relationships directly within the optimization process. Consequently, the probabilistic HFU outputs act not only as classification probabilities, but also as continuously enforced petrophysical constraints that guide permeability prediction during training.

This integrated framework extends conventional reservoir characterization workflows into a modern machine learning paradigm, providing a more flexible, physically guided, and uncertainty-aware approach for permeability prediction within heterogeneous reservoirs under limited-data conditions.

## Geological setting and dataset description

The Shahd SE field is located in the Western Desert of Egypt within the Abu Gharadig Basin, one of the main hydrocarbon-producing basins in the region^29^. The Lower Bahariya Formation represents one of the primary reservoir intervals within the basin and consists predominantly of clastic sediments deposited during the Early Cretaceous^30^. The reservoir is composed mainly of fluvial to shallow-marine sandstones interbedded with shale and siltstone layers, resulting in significant vertical and lateral heterogeneity.

Petrophysically, the Lower Bahariya reservoir exhibits variable reservoir quality due to differences in depositional facies, diagenetic processes, and clay content. Porosity values typically range from moderate to high, while permeability varies over several orders of magnitude depending on pore throat size distribution and grain packing characteristics. These heterogeneities strongly influence fluid flow behavior and present challenges for reliable permeability prediction using conventional log-based methods. Core measurements from the Shahd SE wells indicate the presence of multiple reservoir rock types with distinct pore structure characteristics, making the reservoir an appropriate candidate for hydraulic flow unit analysis and machine learning–based permeability modeling.

**Description of available wells**.

The dataset used in this study includes routine core analysis data from three wells in the Shahd SE field: Shahd SE-05, Shahd SE-32, and Shahd SE-42. These core measurements provide laboratory-derived values of horizontal permeability and effective porosity.

Core measurements used in this study were obtained from Routine Core Analysis (RCAL) and limited Special Core Analysis (SCAL) conducted on samples collected from the three wells. These laboratory measurements provide reliable petrophysical data used for reservoir quality evaluation and permeability model calibration.

The cored intervals are located within the Lower Bahariya reservoir, with total core thickness ranging from 61 ft to 90 ft. Plug samples extracted from these intervals were analyzed to measure porosity and horizontal permeability under both ambient laboratory conditions (RCAL) and overburden pressure conditions (SCAL).

RCAL measurements, which represent the majority of the dataset, include porosity and permeability determined at ambient conditions. A smaller subset of samples was analyzed under overburden pressure to account for the effect of reservoir stress on pore structure and permeability. The distribution of core intervals and the number of analyzed plug samples for each well are summarized in Table 1.

**Table 1:** Summary of core intervals and the number of porosity and permeability measurements at ambient and overburden pressure conditions for the studied wells.

Parameter		Shahd SE-05	Shahd SE-32	Shahd SE-42
Core Interval	From	9431 ft.	9420 ft.	9495 ft.
	To	9513 ft.	9513 ft.	9556 ft.
Core Length		82 ft.	90 ft.	61 ft.
Routine Core Analysis (RCAL)	Ambient conditions	83 plugs	66 plugs	46 plugs
Special Core Analysis (SCAL)	Overburden pressure	15 plugs	9 plugs	11 plugs

**Well log dataset**

The well log dataset used in this study comprises conventional wireline logs available across all wells in the Shahd SE field, including gamma ray (GR), bulk density (RHOB) and neutron porosity (NPHI). In addition, interpreted petrophysical logs, including shale volume (VCL) and effective porosity (PHIE), were incorporated. These were generated through standard petrophysical evaluation workflows and offer enhanced insight into reservoir characterization.

## Methodology

**Fig. 1:**
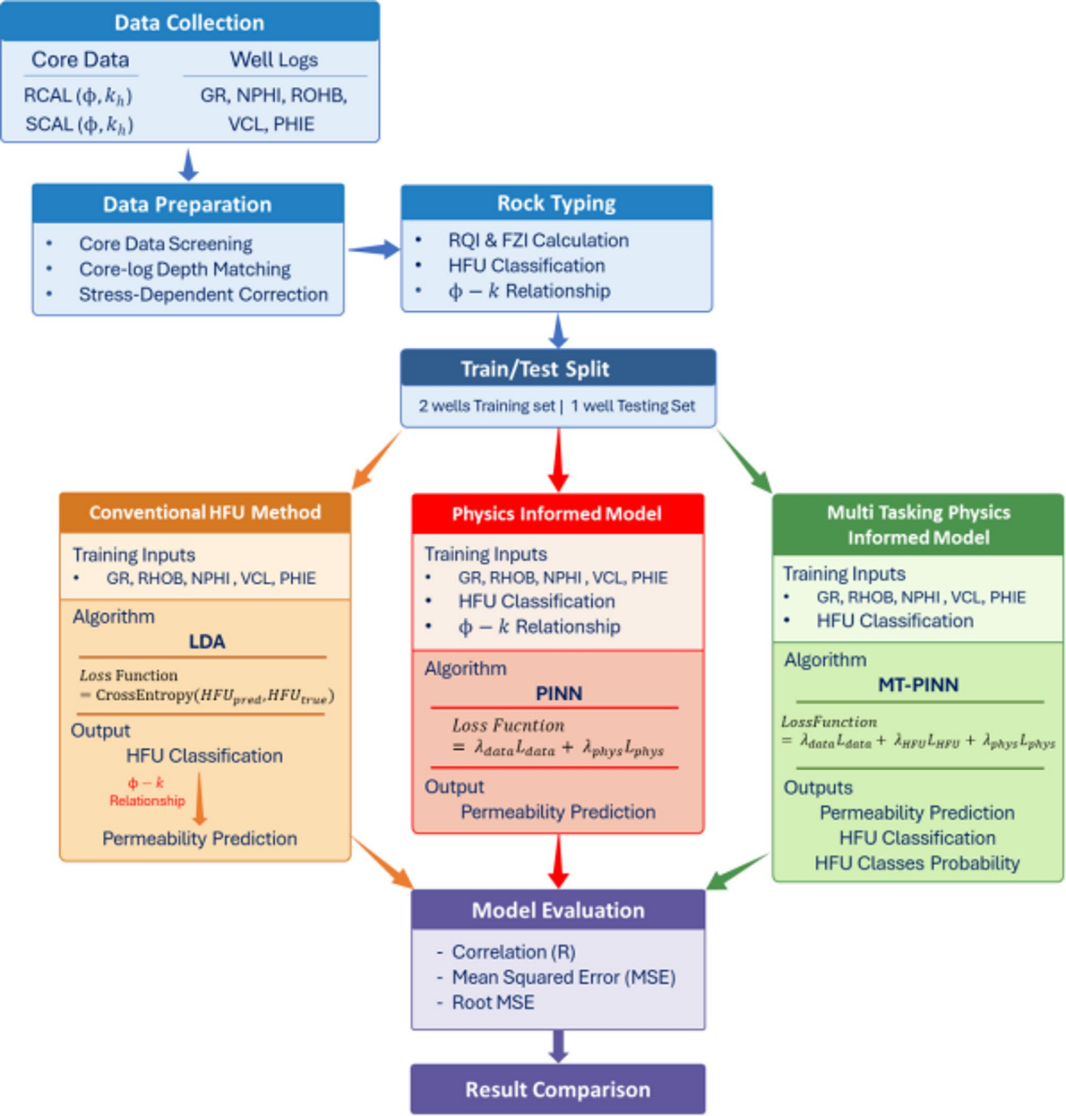
Workflow of permeability prediction using conventional HFU analysis and machine learning methods.

The overall workflow adopted in this study is illustrated in Fig. 1. The process begins with data collection, including core measurements (RCAL and SCAL) and conventional well logs. This is followed by data preparation, where core data are screened, depth-matched with wireline logs, and corrected for stress effects to ensure consistency with reservoir conditions. A petrophysical rock typing framework is then established by computing RQI and FZI, defining hydraulic flow units (HFUs), and deriving porosity–permeability relationships. The dataset is subsequently divided into training and testing wells. Permeability prediction is performed using three approaches: a conventional HFU-based method, a physics-guided neural network (PINN Algorithm), and a multi-task physics-guided neural network (MT-PINN Algorithm). Finally, model performance is evaluated using statistical metrics, and the results are compared to assess the effectiveness of each approach.

### Core data processing and quality control

#### Core data screening

The core dataset was screened to ensure data reliability prior to analysis. Permeability and porosity measurements were checked for unit consistency, depth reference accuracy, and alignment with the target reservoir interval. Samples with missing, placeholder, or physically unrealistic values were removed. Permeability–porosity crossplots were used to identify outliers, while non-reservoir intervals were assigned very low porosity and permeability values to represent tight lithologies. The screened dataset was subsequently used for reservoir quality analysis, hydraulic flow unit identification, and permeability modeling.

#### Depth matching with well logs

Accurate depth alignment between core and wireline logs is essential for reliable petrophysical interpretation. Core depths may differ from log depths due to core expansion, measurement uncertainties, and reference discrepancies, leading to potential errors in core–log integration.

To address this, core gamma-ray measurements were aligned with the wireline gamma-ray (GR) log using a cross-correlation-based approach. Gamma-ray logs were selected due to their consistent lithological response and common use in depth matching. The cross-correlation function is defined as^31^:

$$\:{R}_{xy}\left(k\right)=\:\sum\:_{i=1}^{N}x\left(i\right)y(i+k)$$

where $\:x\left(i\right)$ and $\:y(i+k)$ represent the wireline and core GR measurements, $\:k$ is the lag (shift) applied between the two signals, and N is the number of samples.

#### Stress-dependent correction of RCAL measurements

RCAL measurements, typically acquired at ambient laboratory conditions, were corrected to reservoir conditions using stress-dependent relationships derived from SCAL data. Permeability and porosity decrease with increasing effective stress due to pore compression and throat closure, commonly described by^32^:

$$\:{\upphi\:}\left(\mathrm{P}\right)=\:{{\upphi\:}}_{ref}{e}^{-{C}_{{\upphi\:}}(P-{P}_{ref})}$$

$$\:\mathrm{k}\left(\mathrm{P}\right)=\:{\mathrm{k}}_{ref}{e}^{-{C}_{\mathrm{k}}\left(P-{P}_{ref}\right)}$$

where $\:{{\upphi\:}}_{ref}$ and $\:{\mathrm{k}}_{ref}$ are measured at reference pressure $\:{P}_{ref}$ and $\:{C}_{{\upphi\:}}$ and $\:{C}_{\mathrm{k}}$ are stress-sensitivity coefficients. In this study, measurements at 400 psi were corrected to reservoir pressure (5225 psi) using these relationships. Conventional Reservoir Quality Analysis.

#### Training and testing data split

To ensure a consistent and unbiased evaluation of the proposed models, the dataset was partitioned at the well level into training and testing subsets. Two wells, Shahd SE-05 and Shahd SE-42, were used for model training, while a third well, Shahd SE-32, was reserved as a blind test dataset. Among the three wells, Shahd SE-32 was selected as the primary blind test well presented in the main workflow because it contains a broader distribution of low-, medium-, and high-permeability intervals while also representing the smallest portion of the available dataset (~ 23%). This configuration provides a more challenging and rigorous validation scenario for evaluating model transferability under limited-data conditions. This well-based split strategy prevents data leakage and provides a realistic assessment of model generalization to unseen reservoir conditions. Importantly, the same dataset partitioning was applied consistently across all implemented models, including the classical HFU method, PINN, and MT-PINN frameworks, to ensure a fair and direct comparison of their predictive performance.

The input features used in all models consist of conventional wireline and petrophysical logs commonly associated with reservoir quality, including gamma ray (GR), neutron porosity (NPHI), bulk density (RHOB), shale volume (VCL) and effective porosity (PHIE). These logs provide complementary information related to lithology, clay content, pore structure, and matrix density, which collectively influence the spatial distribution of permeability within the reservoir. Core-derived permeability measurements were used as the target variable for model training and evaluation.

Given that permeability values typically span several orders of magnitude and exhibit a log-normal distribution, the target variable was transformed into logarithmic space during training. This transformation stabilizes variance, reduces the influence of extreme values, and improves the learning efficiency of the models. Model predictions were subsequently transformed back to linear space for comparison with measured core permeability values.

### Petrophysical framework and rock typing

#### Reservoir quality index (RQI) and flow unit framework

The Reservoir Quality Index (RQI) was computed from routine core analysis using horizontal permeability ($\:{k}_{h}$) and effective porosity ($\:{\upphi\:}$) following the formulation proposed by A. A. Amaefule et al. (1993)^6^:

$$\:RQI=0.0314\sqrt{\frac{{k}_{h}}{{\upphi\:}}}$$

RQI provides an estimate of the effective pore throat radius controlling fluid flow. To normalize porosity with respect to the rock matrix, the normalized porosity ($\:{{\upphi\:}}_{z}$) was calculated was calculated, enabling consistent comparison between samples with different porosity values.

The Flow Zone Indicator (FZI), which characterizes pore throat size distribution, was then derived as:

$$\:FZI=\frac{RQI}{{{\upphi\:}}_{z}\text{}}$$

Based on FZI, Hydraulic Flow Units (HFUs) were identified as rock groups with similar pore throat geometry and consistent flow behavior. This classification reduces heterogeneity and improves the predictability of permeability–porosity relationships.

#### Porosity–permeability relationship within HFUs

Within each HFU, permeability–porosity relationships were established using core data. Given the log-normal distribution of permeability, regression was performed on log-transformed permeability and expressed using a power-law model:

$$\:k=a{{\upphi\:}}^{b}$$

where $\:k$ is permeability (mD), $\:{\upphi\:}$ is effective porosity (fraction), and $\:a$ and $\:b$ are empirical coefficients determined through regression analysis.

#### Log-based rock type prediction

To extend HFU classification to uncored intervals, the rock type classification was performed using Linear Discriminant Analysis (LDA), originally introduced by Fisher (1936), which seeks linear combinations of variables that maximize class separability^33^. Core-derived HFU labels were matched with corresponding log measurements during training. The input features included GR, NPHI, RHOB, VCL and PHIE, capturing lithology, clay content, and pore characteristics. Linear Discriminant Analysis (LDA) was selected in this study due to its relatively low model complexity, interpretability, and reduced susceptibility to overfitting when applied to limited datasets. Given the relatively small number of available wells and core samples, the use of simpler linear classifiers was considered more appropriate for generating stable probabilistic HFU predictions.

During the training stage, core samples with known rock type labels were matched with the corresponding well log measurements at the same depths. The LDA algorithm then estimated discriminant functions of the form:

$$\:{D}_{k}=\:{b}_{k}\:+\:{w}_{1k}\:GR\:+{w}_{2k}\:RHON\:+\:{w}_{3k}\:NPHI+{w}_{5k}\:PHIE\:+\:{w}_{6k}\:VCL\:$$

where $\:{D}_{k}$ represents the discriminant score for rock type $\:k$, and $\:w$ and $\:b$ are coefficients determined during model training. For each depth sample, the rock type was assigned to the class with the highest discriminant score.

#### Permeability estimation using HFUs

Permeability was estimated by combining log-derived porosity with the predicted HFU classification using the corresponding HFU-specific porosity–permeability relationships^6,34^. This workflow enables continuous permeability prediction along the wellbore and represents a standard petrophysical approach linking core measurements to well log data.

### Physics guided modeling approaches

#### Physics guided neural network (PINN algorithm) modeling

To incorporate physical knowledge into the permeability prediction process, a physics-informed neural network (PINN) model was developed. Unlike purely data-driven approaches, the PINN framework integrates established petrophysical relationships into the training process by augmenting the loss function with a physics-based constraint. The PINN methodology was originally introduced by Raissi et al. (2019), who demonstrated the integration of governing physical laws into neural network training^35^. In the present study, the term “physics-guided” is used to describe the incorporation of empirical petrophysical consistency constraints within the neural-network optimization process rather than direct enforcement of governing flow partial differential equations such as Darcy flow or mass conservation. Accordingly, the proposed framework follows the broader PINN philosophy of integrating physics-related constraints into the training process while remaining distinct from classical PDE-constrained PINN implementations. Given an input feature vector $\:X={[GR,\:NPHI,\:VCL,\:PHIE,\:RHOB]}^{T}$, the neural network learns a mapping:

$$\:X\:\to\:\:\widehat{K}$$

where $\:\widehat{K}$ is the predicted permeability. In this study, the permeability–porosity relationship derived from HFU analysis is used to guide the model predictions and is expressed as:

$$\:{K}_{phy}=\:{a}_{r}\:{{\upphi\:}}^{{b}_{r}}$$

where $\:{\upphi\:}$ is porosity and $\:{a}_{r}$ and $\:{b}_{r}$ are rock-type-dependent coefficients associated with the known hydraulic flow unit $\:r$. Since rock types are predefined, the physics constraint is deterministic.

The training objective combines a data loss and a physics loss. The data loss ensures agreement with measured permeability:

$$\:{L}_{data}=\:\frac{1}{N}\:\sum\:_{i=1}^{N}{({\widehat{K}}_{i}-\:{K}_{i})}^{2}$$

This formulation ensures agreement between predicted and measured permeability. To enforce consistency with the HFU-derived relationship, a physics-based constraint is introduced in logarithmic space:

$$\:{L}_{phys}=\:\frac{1}{N}\:\sum\:_{i=1}^{N}log{\left({\widehat{K}}_{i}\right)-\:\mathrm{l}\mathrm{o}\mathrm{g}\left({K}_{phys,i}\right)}^{2}$$

This formulation accounts for the wide dynamic range of permeability and stabilizes the training process. These components are combined into a unified loss function:

$$\:L=\:{\lambda\:}_{data}{L}_{data}+\:{\lambda\:}_{phys}{L}_{phys}$$

where $\:{\lambda\:}_{data}$ and $\:{\lambda\:}_{phys}$ are weighting parameters that control the contribution of each term.

This formulation enables the model to combine data-driven learning with physical consistency. However, its reliance on predefined rock type labels limits its ability to represent transitional facies behavior and introduces sensitivity to classification errors.

#### Multi-task physics guided neural network (MT-PINN algorithm) modeling

To address the limitations of explicit rock type classification, a multi-task physics-guided neural network (MT-PINN) was developed. The model extends the PINN framework by jointly predicting permeability and rock type probabilities. For an input vector $\:X$, the model learns a joint mapping:

$$\:X\:\to\:\left\{\widehat{p},\:\widehat{K}\right\}$$

where $\:\widehat{K}$ is the predicted permeability and $\:\widehat{p}=[{p}_{1},\:{p}_{2},\:\dots\:,\:{p}_{R}]$ represents the probability distribution over $\:R$ rock types. These probabilities are obtained using a softmax function, allowing a continuous representation of facies.

Instead of assigning a single rock type, the model predicts probabilities using a softmax function:

$$\:{p}_{r}=\:\frac{\mathrm{e}\mathrm{x}\mathrm{p}\left({z}_{r}\right)}{{\sum\:}_{j=1}^{R}\mathrm{e}\mathrm{x}\mathrm{p}\left({z}_{j}\right)},\:\:\:\:\:r=1,\dots\:,R$$

where $\:{z}_{r}$ are the logits produced by the network. The overall permeability is then computed as a weighted combination:

$$\:{K}_{phy}=\:\sum\:_{r=1}^{R}pr\:\left({a}_{r}\:{{\upphi\:}}^{{b}_{r}}\right)$$

where $\:{a}_{r}$ and $\:{b}_{r}$ are the HFU-specific fitting parameters and $\:{\upphi\:}$ represents porosity.

The proposed probability-weighted formulation should not be interpreted as a rigorous physical upscaling or volumetric averaging scheme for heterogeneous porous media. Instead, the formulation is intended as a soft probabilistic transition mechanism between competing HFU-dependent porosity–permeability relationships within the learning framework. The weighted combination allows the model to represent uncertainty and gradual transitions between hydraulic flow units while avoiding abrupt permeability discontinuities commonly associated with hard rock-type classification.

In the present study, an arithmetic probability-weighted formulation was adopted primarily for computational simplicity, numerical stability, differentiability, and compatibility with gradient-based neural-network optimization. Alternative averaging schemes, including harmonic or geometric averaging, may provide different representations of heterogeneous flow behavior and could be explored in future investigations depending on the target scale of permeability modeling and flow characterization objectives.

This formulation effectively transforms the classical HFU workflow into a continuous and differentiable framework, replacing hard classification with a soft probabilistic representation.

The training objective consists of three complementary components. The data loss minimizes the discrepancy between predicted and measured permeability:

$$\:{L}_{data}=\:\frac{1}{N}\:\sum\:_{i=1}^{N}{({\widehat{K}}_{i}-\:{K}_{i})}^{2}$$

The rock type classification loss improves the accuracy of predicted facies:

$$\:{L}_{RT}=\:\frac{1}{N}\:\sum\:_{i=1}^{N}\sum\:_{r=1}^{R}{y}_{i,\:\:r}\:log\left({p}_{i,r}\right)$$

where $\:{y}_{i,\:\:r}$ represents the true rock type labels. Finally, the physics loss enforces consistency between predicted permeability and the underlying petrophysical relationships, applied in logarithmic space to account for the wide dynamic range of permeability:

$$\:{L}_{phys}=\:\frac{1}{N}\:\sum\:_{i=1}^{N}log{\left({\widehat{K}}_{i}\right)-\:\mathrm{l}\mathrm{o}\mathrm{g}\left({K}_{phys,i}\right)}^{2}$$

These components are combined into a unified loss function:

$$\:L=\:{\lambda\:}_{data}{L}_{data}+\:{\lambda\:}_{RT}{L}_{RT}+\:{\lambda\:}_{phys}{L}_{phys}$$

where $\:{\lambda\:}_{data}$, $\:{\lambda\:}_{RT}$ and $\:{\lambda\:}_{phys}$ weighting coefficients controlling the relative contribution of each loss component during training.

By jointly optimizing permeability prediction, probabilistic HFU classification, and petrophysical consistency constraints, the proposed MT-PINN framework integrates data-driven learning and probabilistic reservoir characterization within a unified workflow. In addition to predicting permeability, the model simultaneously outputs both discrete HFU classifications and their associated class probabilities, enabling deterministic and probabilistic interpretation of reservoir quality while reducing sensitivity to hard HFU boundaries and classification uncertainty.

The final MT-PINN architecture and training configuration adopted in this study are summarized in Table 2.

**Table 2:** MT-PINN architecture and training configuration.

Component	Configuration
Input features	GR, VCL, NPHI, RHOB, PHIE
Number of input features	5
Network architecture	Multi-task fully connected neural network
Shared hidden layers	2
Neurons per hidden layer	64
Activation function	ReLU
HFU classification branch	Fully connected classification layer
Permeability prediction branch	Fully connected regression layer predicting log(Kh)
Number of HFU classes	6
Output activation (HFU branch)	Softmax
Approximate trainable parameters	4,999 (~ 5,000)
Optimizer	Adam
Learning rate	0.001
Batch strategy	Full-batch training
Number of epochs	500
Random seed	42
Framework	PyTorch
Permeability loss	Mean squared error (MSE)
HFU classification loss	Cross-entropy loss
Physics-guided constraint	Probability-weighted HFU porosity–permeability relationship
Loss weights	$\:{\lambda\:}_{data}=0.3,\:{\lambda\:}_{RT}=0.4,\:{\lambda\:}_{phys}=0.2$
Hyperparameter optimization	Grid-search sensitivity analysis

No explicit regularization techniques such as dropout, weight decay, or early stopping were applied in the present study due to the intentionally compact network architecture and relatively limited number of trainable parameters. Instead, model stability was supported through the integration of probabilistic HFU representations and petrophysical constraints, which provided an implicit constraint on physically consistent permeability behavior during training.

By jointly optimizing these objectives, the MT-PINN model integrates data-driven learning, physical constraints, and probabilistic rock typing within a unified framework. To evaluate the sensitivity of the MT-PINN framework to loss-function weighting, a grid-search analysis was performed using different combinations of the regression, classification, and physics-based loss coefficients $\:{L}_{data}$, $\:{L}_{RT}$, and $\:{L}_{phys}$.

In addition to predicting permeability, the model simultaneously outputs both discrete rock type classifications and their associated class probabilities, providing a measure of prediction confidence. This dual representation enables the use of either deterministic or probabilistic porosity–permeability relationships, thereby reducing sensitivity to classification errors and allowing smoother transitions between flow units. Consequently, the model offers a more realistic representation of reservoir heterogeneity compared to both conventional HFU-based methods and standard PINN approaches.

### Model validation evaluation metrics

The predictive performance of the permeability models was evaluated by comparing predicted permeability values with measured core permeability data from the testing dataset. Several statistical metrics were used to quantify the agreement between predicted and observed values.

The Pearson correlation coefficient ($\:R$) was used to evaluate the strength of the relationship between predicted and measured permeability values in logarithmic space, which is appropriate given the wide dynamic range of permeability. Higher values of $\:R$ indicate stronger agreement between predictions and observations across different orders of magnitude.

In addition, the root mean squared error ($\:RMSE$) was calculated in linear space to quantify absolute prediction errors, providing an interpretable measure in the same units as permeability. To further assess model performance across varying permeability scales, the root mean squared error in logarithmic space ($\:{RMSE}_{log}$) was also computed, offering a scale-independent evaluation of prediction accuracy.

## Results

**Core data screening**.

Prior to reservoir quality analysis and permeability modeling, the core dataset was examined to identify incomplete or unreliable measurements. During this screening process, three RCAL samples were excluded from the dataset because either porosity or permeability measurements were missing due to broken core plugs or laboratory processing failures, resulting in incomplete paired measurements. These samples could not be used in permeability–porosity analysis because the absence of one parameter prevents reliable evaluation of reservoir quality relationships.

**Fig. 2:**
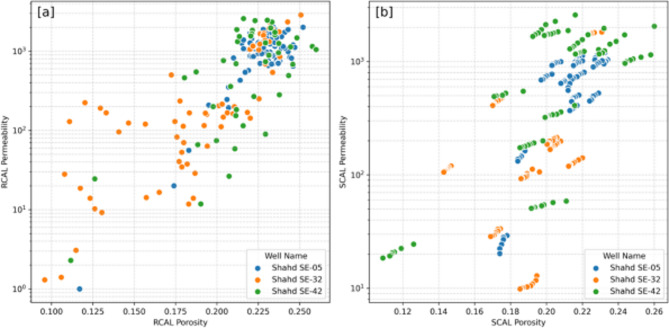
Crossplots of core porosity versus permeability used for data quality assessment. [**a**] RCAL porosity–permeability relationship measured at ambient laboratory conditions. [**b**] SCAL porosity–permeability relationship measured under reservoir pressure conditions.

The distribution of the remaining core measurements is illustrated in Fig. 2. Panel [a] presents the crossplot of RCAL porosity versus permeability measured at ambient laboratory conditions, showing the general permeability–porosity trend within the reservoir. Panel [b] shows the corresponding SCAL porosity–permeability relationship measured under reservoir pressure conditions, which provides a more representative characterization of in-situ reservoir flow properties. Both plots demonstrate a consistent positive relationship between porosity and permeability, although noticeable scatter is present, reflecting variations in pore throat geometry and reservoir heterogeneity.

Non-reservoir intervals were also identified based on conventional and petrophysical log interpretation. To represent these tight lithologies within the dataset, 20 synthetic samples were generated at selected depths corresponding to non-reservoir intervals. In practical reservoir characterization workflows, non-reservoir intervals are commonly observed in well logs but are rarely selected for detailed core permeability measurements due to their negligible porosity and permeability values. Consequently, relying solely on measured core data may lead to underrepresentation of extremely low-permeability and non-reservoir conditions within the training dataset.

The generated samples were therefore introduced to provide physically realistic representation of tight non-reservoir intervals encountered along the logged sections of the studied wells. These samples were not randomly generated, but were constrained using realistic low-porosity and ultra-low permeability ranges consistent with non-reservoir behavior observed in the studied formation. The primary objective of this augmentation was not to artificially improve predictive performance, but rather to reduce extrapolation instability and ensure that the probabilistic HFU framework was exposed to the full range of geological conditions encountered within the well logs, including non-reservoir intervals. The number of synthetic samples remained limited relative to the full dataset in order to minimize potential bias in the learned porosity–permeability relationships and overall model behavior.

After these quality control and data augmentation steps, the screened core dataset was used for subsequent reservoir quality analysis, hydraulic flow unit identification, and permeability prediction modeling.

**Depth matching with well logs**.

The cross-correlation analysis successfully identified the optimal depth shift required to align the core gamma-ray measurements with the corresponding wireline logs for each well. Table 3 summarizes the calculated lag values, the resulting depth shifts, and the improvement in correlation coefficients obtained after applying the depth correction.

**Table 3:** Cross-correlation depth matching results for the studied wells, showing the optimal lag, calculated depth shift, and correlation coefficients before and after correction.

Well	Optimal Lag (samples)	Depth Shift (ft)	Correlation Before Shift	Correlation After Shift
Shahd SE-05	10	5.0	−0.057	0.713
Shahd SE-32	−1	−0.5	0.902	0.927
Shahd SE-42	28	14.0	−0.029	0.875

**Fig. 3:**
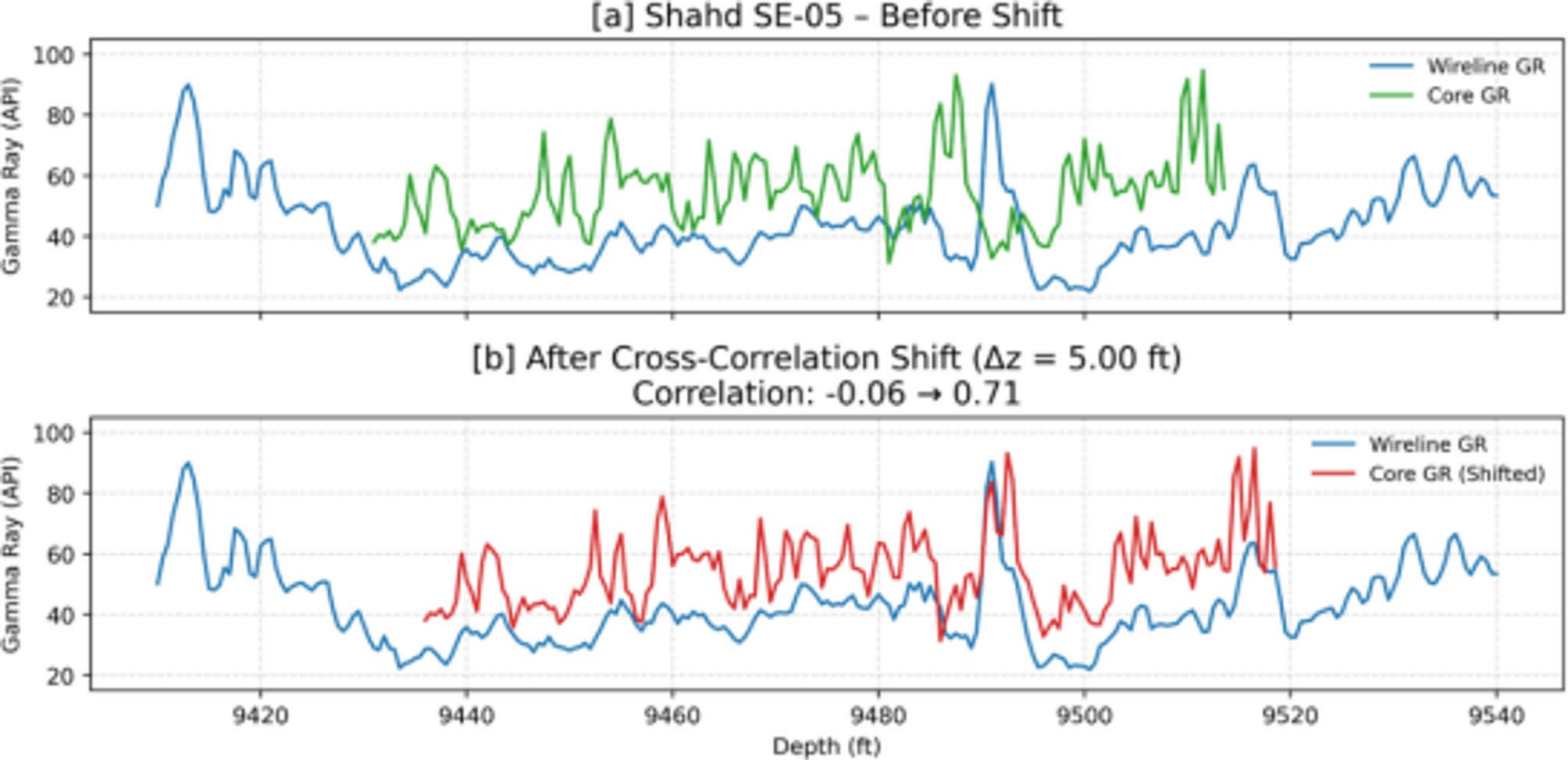
Wireline and core gamma-ray (GR) curves for the Shahd SE-05 well before (**a**) and after (**b**) depth matching using cross-correlation. The applied shift of 5 ft improves the alignment between the two curves.

Figure 3 illustrates an example of the depth matching procedure for the Shahd SE-05 well, showing the wireline and core gamma-ray curves before and after applying the calculated shift. After correction, the major gamma-ray features become well aligned, indicating a successful depth match.

**Verification of the Stress-Correction Model**.

**Fig. 4:**
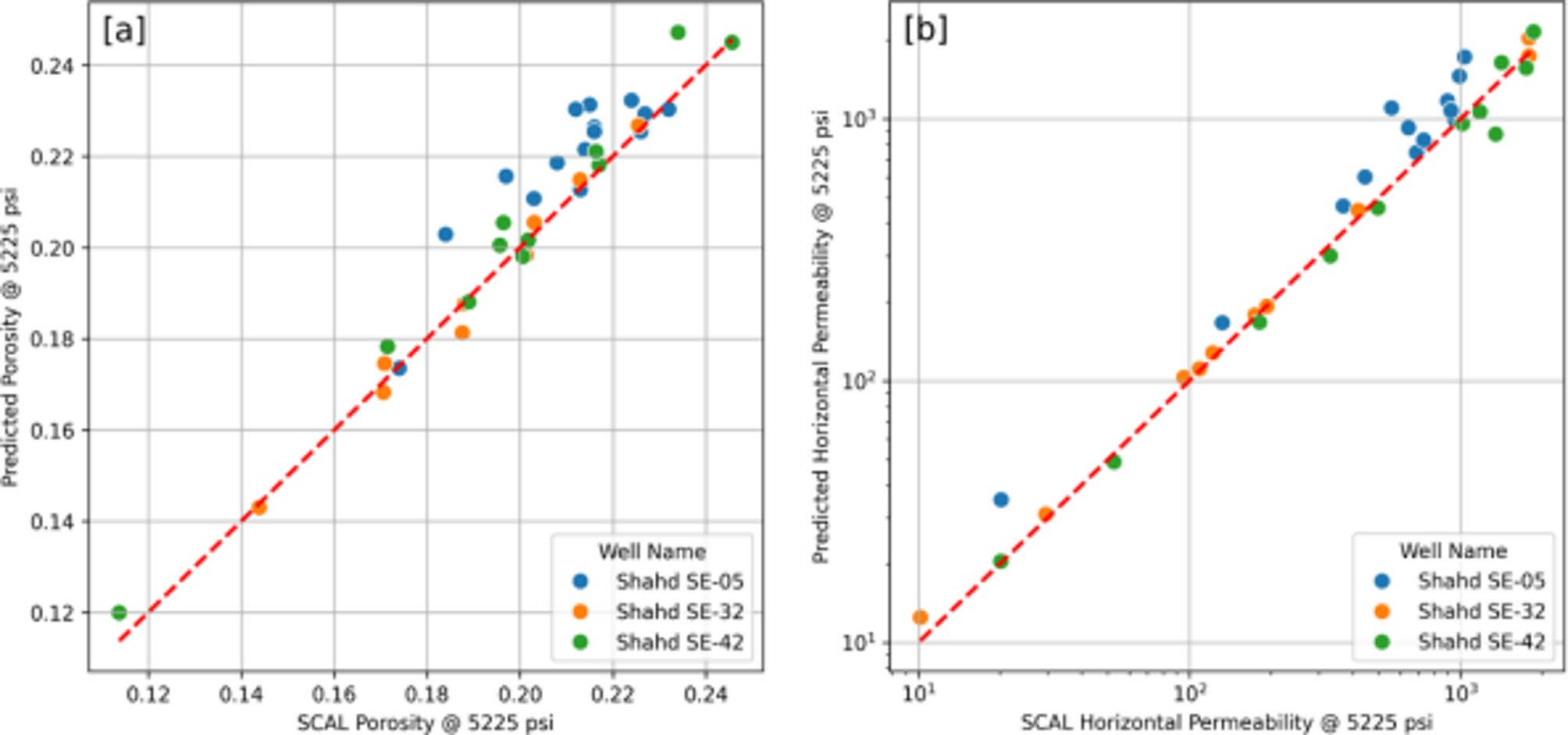
Validation of the traditional stress-correction model at reservoir pressure (5225 psi). [**a**] Crossplot of predicted versus SCAL porosity values and [**b**] Crossplot of predicted versus SCAL permeability values (log scale). The dashed line represents the 1:1 relationship.

To verify the reliability of the stress-correction model, the predicted porosity and permeability values at reservoir pressure were compared with SCAL measurements. Because SCAL measurements were obtained at discrete pressure levels, the measured values were interpolated to the reservoir pressure of 5225 psi to provide reference values for comparison. Figure 4 shows crossplots of predicted versus SCAL measurements at reservoir pressure. Panel (a) presents the comparison for porosity, while panel (b) shows the comparison for permeability.

The results demonstrate strong agreement between predicted and measured values. The porosity predictions achieved an RMSE of 0.008 with an R² of 0.904, indicating that the stress-correction model effectively reproduces the pressure-dependent behavior of porosity. For permeability, the model achieved an RMSE of 200.09 mD and an R² of 0.962, demonstrating strong predictive capability.

**Hydraulic Flow Unit (HFU) identification**.

**Fig. 5:**
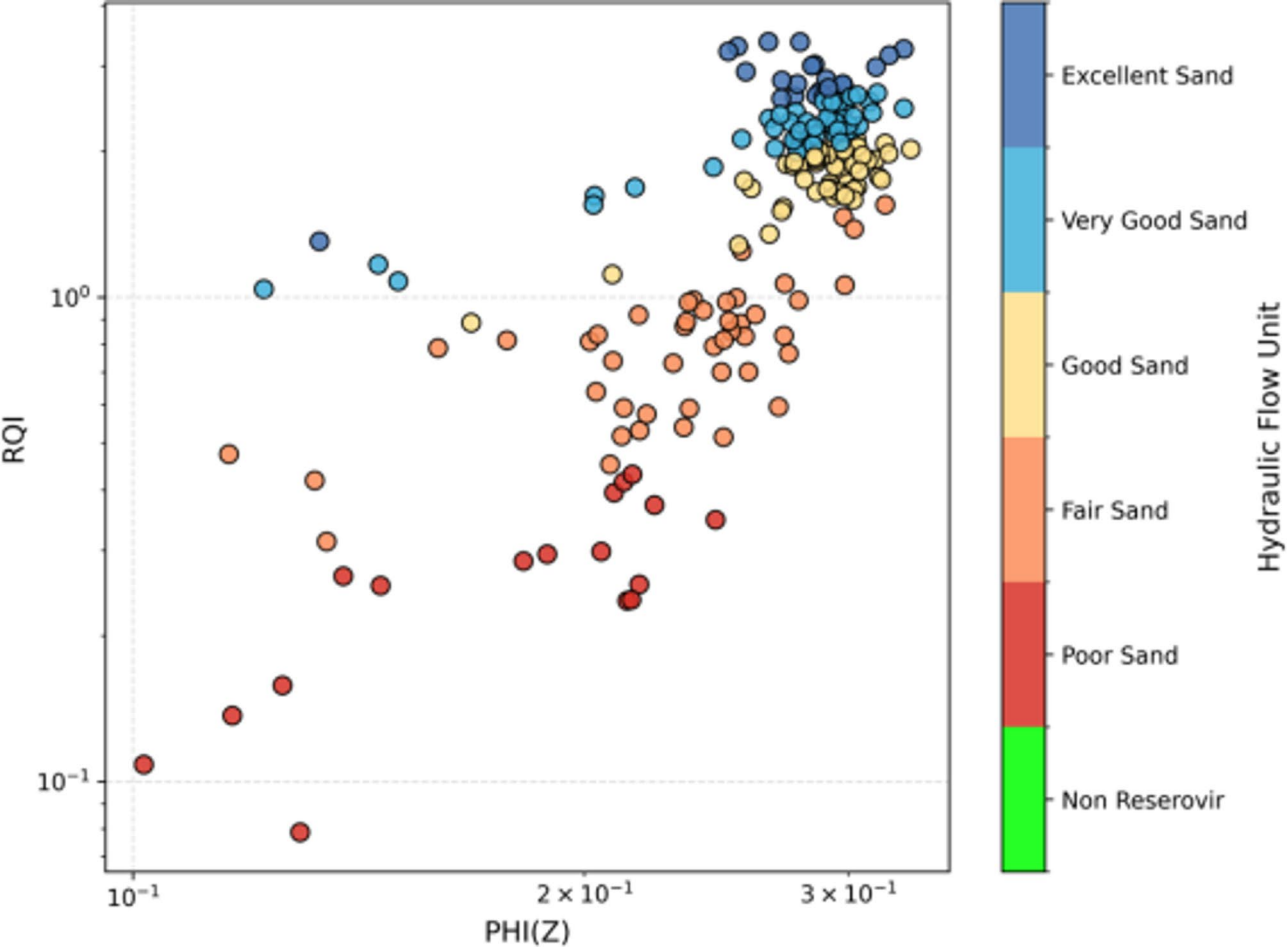
Crossplot of reservoir quality index ($\:\boldsymbol{R}\boldsymbol{Q}\boldsymbol{I}$) versus $\:{\boldsymbol{\phi\:}}_{\boldsymbol{z}}$ showing the classification of hydraulic flow units (HFUs). The data points are color-coded according to rock quality classes ranging from non-reservoir to excellent sand.

Core samples from the three studied wells were plotted on the $\:{\mathrm{R}\mathrm{Q}\mathrm{I}-\:{\upphi\:}}_{z}$ crossplot, and clusters of data points exhibiting similar trends were grouped to define distinct hydraulic flow units (HFUs). Each identified HFU represents a reservoir rock type characterized by relatively consistent pore throat geometry and similar fluid flow behavior. The classification of hydraulic flow units was performed based on the Flow Zone Indicator (FZI) values derived from the core measurements (Fig. 5).

Six reservoir quality classes were defined using the following FZI thresholds: Non reservoir ($\:FZI\:<\:0.5$), Poor Sand ($\:0.5\:\le\:FZI\:<\:2$), Fair Sand ($\:2\:\le\:\:FZI\:<\:5$), Good Sand ($\:5\:\le\:\:FZI\:<\:7$), Very Good Sand ($\:7\:\le\:\:FZI\:<\:9$), and Excellent Sand ($\:FZI\:\ge\:\:9$). These classes represent increasing pore throat size and improved reservoir flow capacity.

**Fig. 6:**
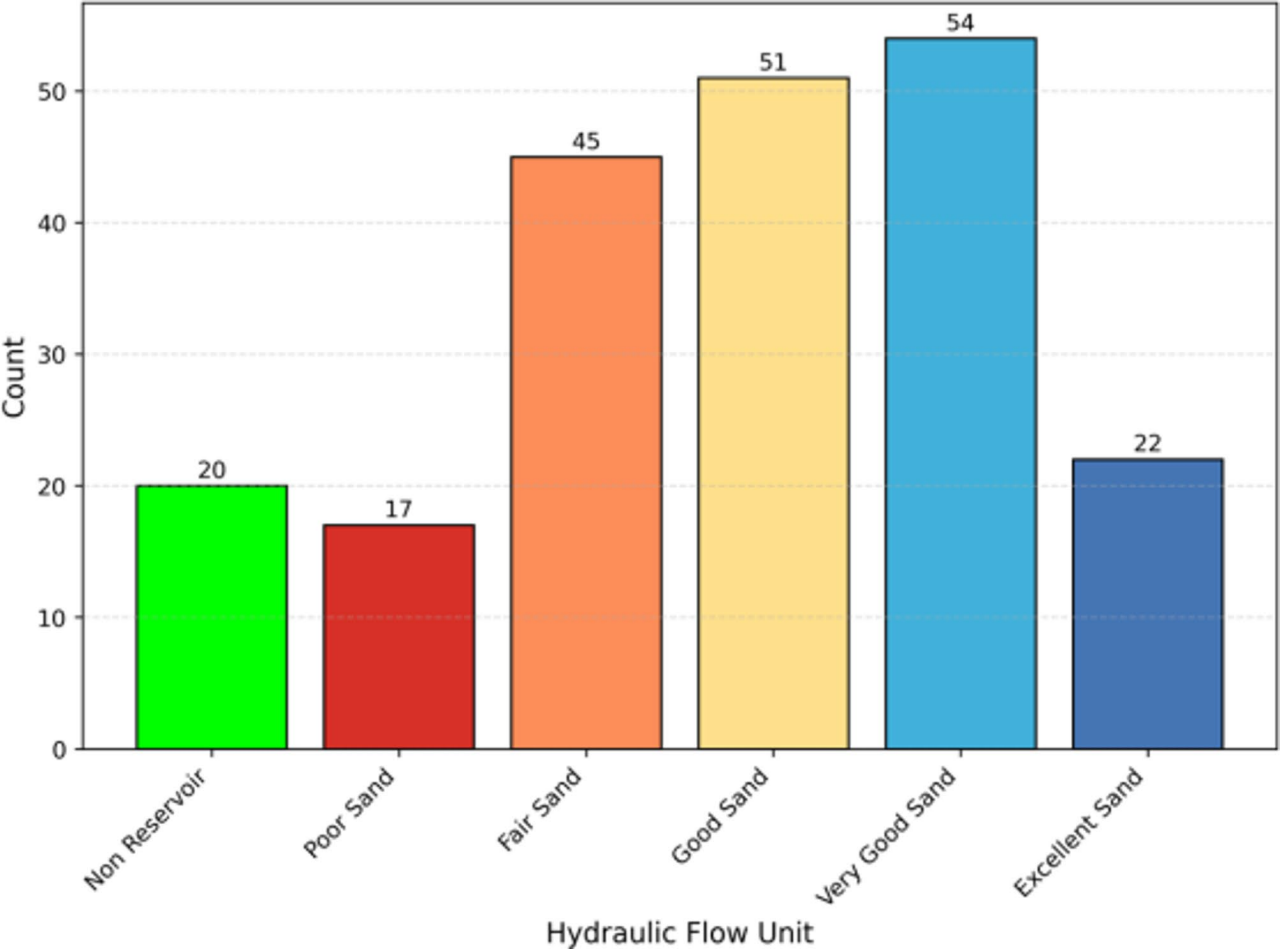
Distribution of core samples across the identified hydraulic flow units based on Flow Zone Indicator (FZI) classification.

The distribution of core samples among the identified HFU is shown in Fig. 6, which illustrates the relative frequency of each reservoir quality class. The results indicate that the majority of the core samples fall within the Good Sand and Very Good Sand categories, comprising 51 and 54 samples, respectively. The Fair Sand class includes 45 samples, while Excellent Sand and Poor Sand represent smaller proportions of the dataset with 22 and 17 samples, respectively, in addition to 20 synthetic samples representing the non reservoir intervals. This distribution suggests that the Lower Bahariya reservoir interval in the studied wells is dominated by moderate to high-quality reservoir facies with well-developed pore systems.

**Fig. 7:**
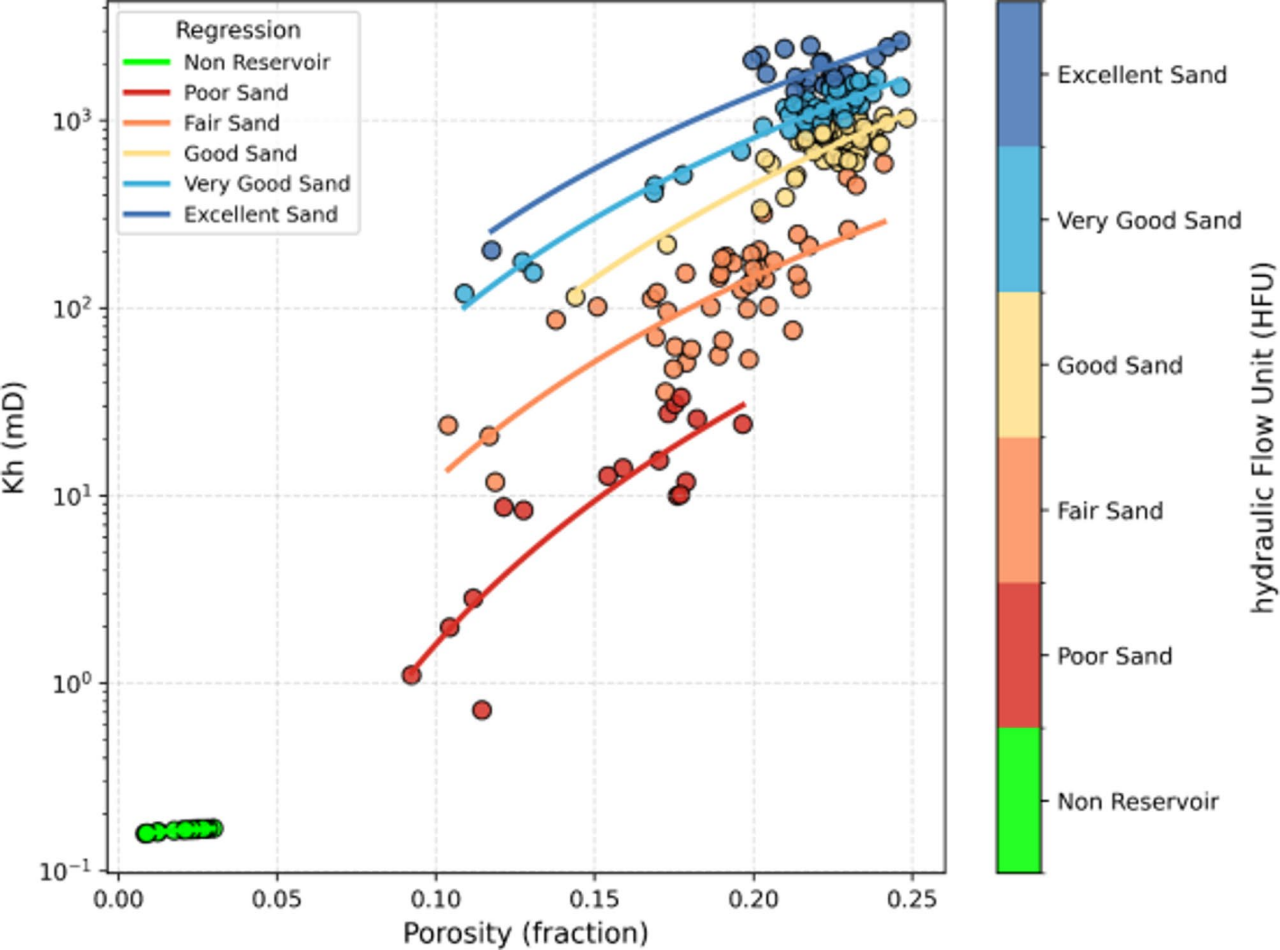
Crossplot of core porosity versus horizontal permeability colored by reservoir rock type, showing the power-law regression relationships derived for the identified hydraulic flow units. Each curve represents the best-fit permeability–porosity relationship for a specific reservoir quality class ranging from Poor Sand to Excellent Sand.

The validity of the HFU classification is further supported by the distinct porosity–permeability relationships observed within the identified rock types. As illustrated in Fig. 7, permeability increases systematically with improving reservoir quality, and separate power-law correlations of the form $\:k=a{{\upphi\:}}^{b}$ were derived for each hydraulic flow unit. The regression results summarized in Table 4 demonstrate that the strength of the porosity–permeability relationship varies among the different rock types, reflecting variations in pore throat geometry and reservoir flow capacity. The Very Good Sand class exhibits the strongest correlation ($\:R^{2}\:=\:0.949$), indicating a relatively uniform pore system with well-connected pore throats. In contrast, the Fair Sand and Excellent Sand classes show weaker correlations, suggesting greater heterogeneity in pore structure and permeability distribution. The Poor Sand and Good Sand classes display moderate correlations, indicating that while permeability generally increases with porosity, additional factors such as grain sorting, clay content, and pore throat variability influence the flow behavior. These results confirm that the HFU classification effectively captures variations in reservoir quality and provides a reliable framework for permeability prediction.

**Table 4:** Porosity–permeability relationships for different reservoir hydraulic flow unit classes. The table lists the fitted power-law equations and the corresponding R² values used to evaluate the strength of the correlation between porosity and permeability within each hydraulic flow unit.

HFU	Equation	*R*²	Interpretation
Non Reservoir	$\:k=0.2{{\upphi\:}}^{0.05}$	1.00	Perfect correlation
Poor Sand	$\:k=35317.11{{\upphi\:}}^{4.34}$	0.769	Moderate correlation
Fair Sand	$\:k=4$60225.87$\:{{\upphi\:}}^{3.725}$	0.639	Weak–moderate correlation
Good Sand	$\:k=$275,510$\:{{\upphi\:}}^{3.98}$	0.766	Moderate correlation
Very Good Sand	$\:k=$208738.5$\:{{\upphi\:}}^{3.456}$	0.949	Strong correlation
Excellent Sand	$\:k=$221496.1$\:{{\upphi\:}}^{3.215}$	0.903	Strong correlation

**Hydraulic Flow Unit Prediction from Well Logs**.

The trained LDA model was applied to the log dataset to predict hydraulic flow units along the wells included in the study. The coefficients of the linear discriminant functions derived from the training dataset are summarized in Table 5. These coefficients define the linear combinations of well log variables used to compute the discriminant score for each hydraulic flow unit. For a given depth sample, the hydraulic flow unit is assigned to the class associated with the highest discriminant score.

**Table 5:** Linear Discriminant Analysis coefficients used for HFU prediction.

HFU	Intercept	GR	NPHI	RHOB	PHIE	VCL
Non Reservoir	531.87	0.239	156.023	−137.35	−1829.82	−98.752
Poor Sand	5.153	0.014	−10.755	3.705	−84.275	0.30
Fair Sand	−13.011	−0.039	−39.895	0.671	72.416	17.519
Good Sand	−76.129	0.049	−14.128	14.55	195.035	4.821
Very Good Sand	−64.190	−0.044	−0.362	12.732	161.631	7.097
Excellent Sand	−44.603	−0.163	−7.050	9.496	121.917	10.910

**Fig. 8:**
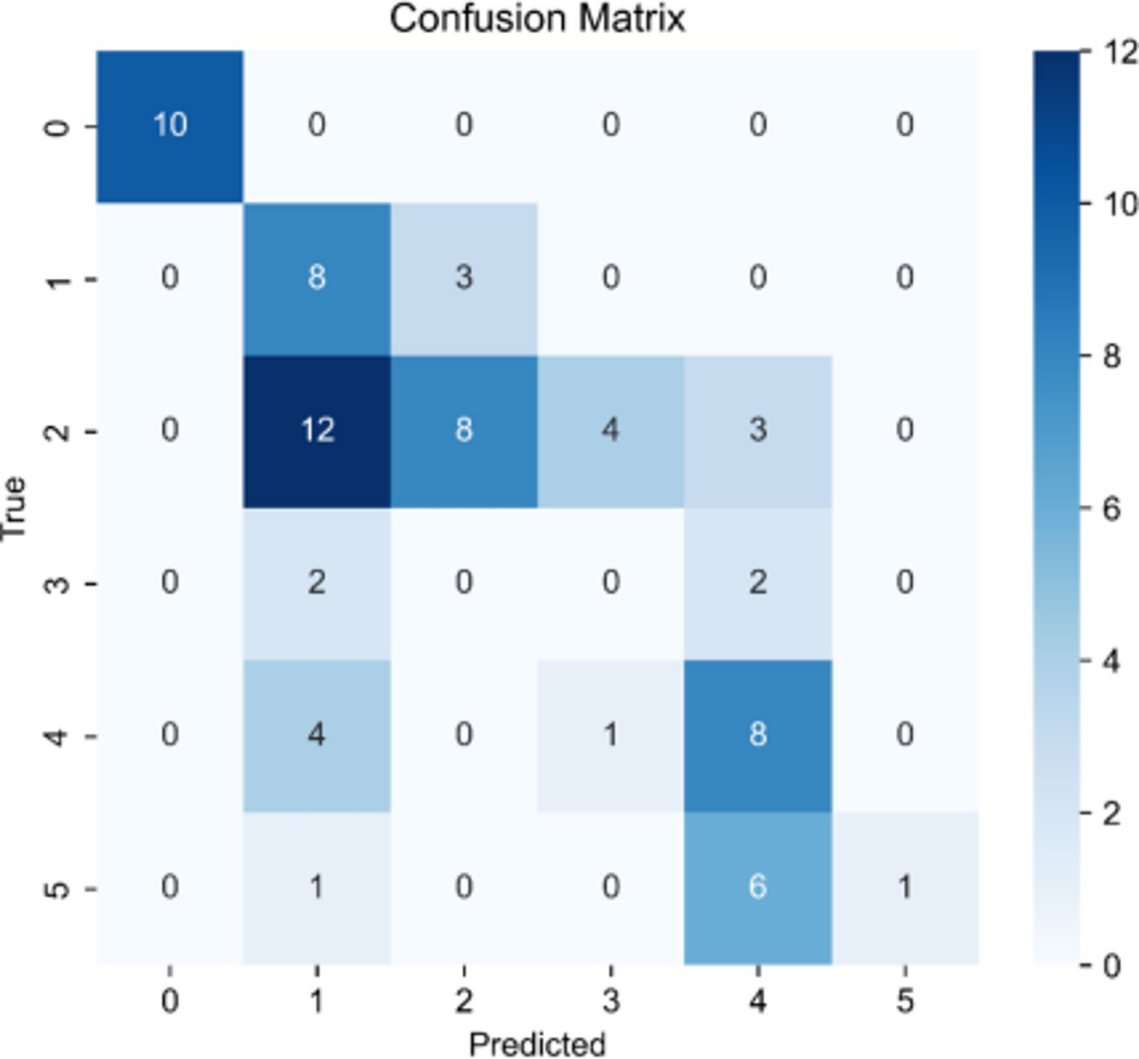
Confusion matrix of the LDA-based hydraulic flow unit (HFU) classification model.

The classification performance of the LDA model demonstrates moderate predictive capability, with an overall accuracy of 48% and a macro-averaged F1-score of 0.47, indicating limited generalization across the six hydraulic flow units (HFUs). The detailed classification metrics are summarized in Table 6, while the corresponding confusion matrix is presented in Fig. 8. Class 0 was perfectly identified due to its distinct non-reservoir petrophysical characteristics. In contrast, substantial overlap was observed among the remaining HFU classes, particularly between Classes 1, 2, and 4, indicating difficulty in separating intermediate reservoir qualities using linear decision boundaries. Class 3 exhibited very weak predictive performance, likely due to limited training samples and overlapping feature distributions. Similarly, several Class 5 samples were incorrectly classified as Class 4, reflecting gradual transitions between higher-quality reservoir intervals.

**Table 6:** LDA-based HFU classification performance.

Class	Precision	Recall	F1-score	Support
0	1.00	1.00	1.00	10
1	0.30	0.73	0.42	11
2	0.73	0.3	0.42	27
3	0	0	0	4
4	0.42	0.62	0.50	13
5	1.00	0.12	0.22	8
Accuracy			0.48	73
Macro Avg	0.64	0.48	0.47	73

Overall, the results highlight the limitations of deterministic LDA-based HFU classification in heterogeneous carbonate reservoirs characterized by class imbalance, limited sample size, and overlapping well-log responses. These limitations further motivate the adoption of probabilistic HFU representations within the proposed MT-PINN framework.

**Hydraulic Flow Unit Permeability Prediction**.

**Fig. 9:**
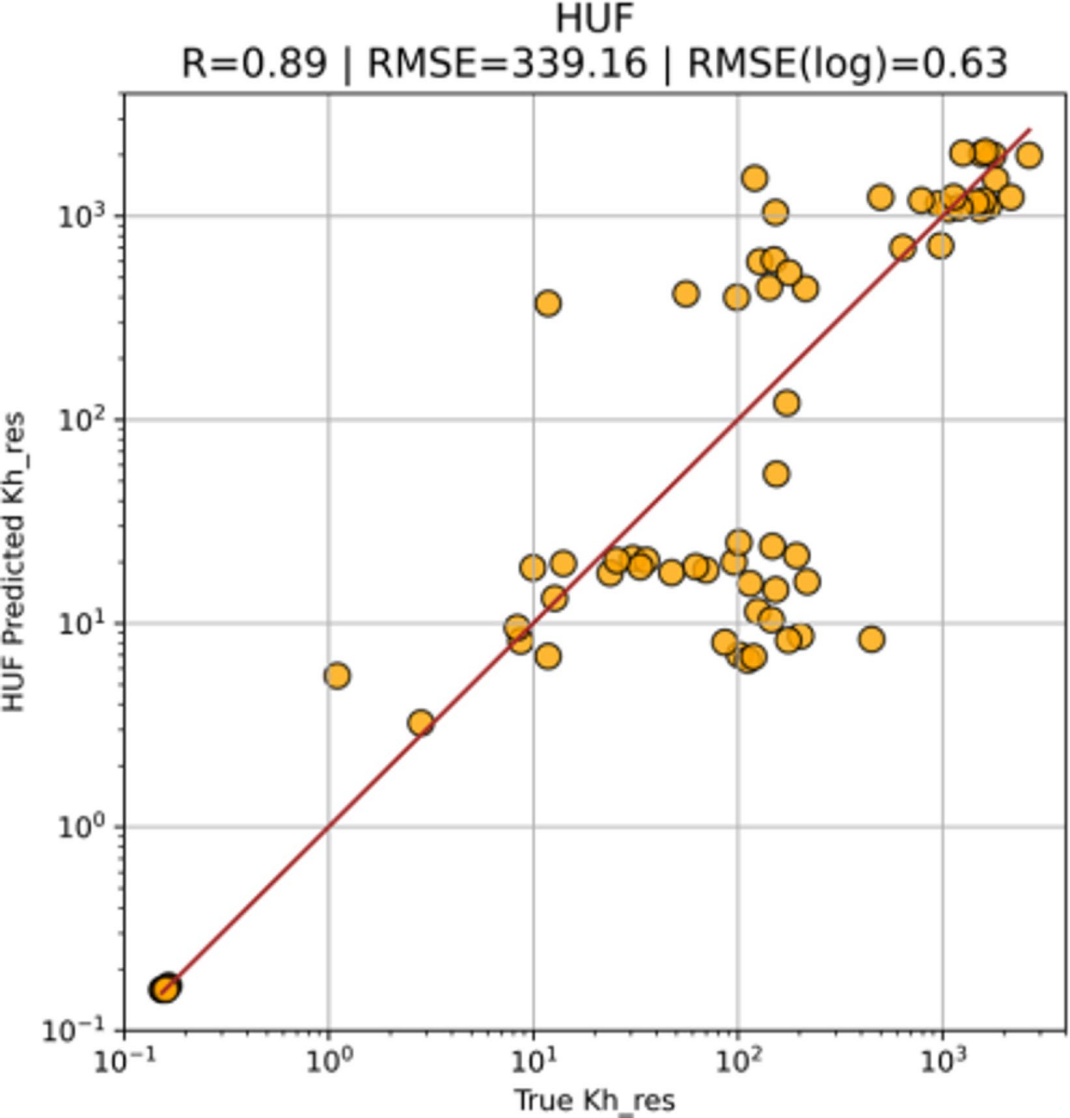
Crossplot of measured core permeability versus permeability predicted using the classical hydraulic flow unit (HFU) method. The brown line represents the 1:1 reference line.

Permeability predictions obtained using the classical HFU-based approach were compared with measured core permeability values. Figure 9 illustrates the relationship between predicted and measured permeability on the test set using the rock-type-dependent porosity–permeability correlations.

The crossplot of measured versus HFU-predicted permeability demonstrates a generally strong correlation, with a correlation coefficient of $\:R\:=\:0.89$, indicating that the HFU-based approach is capable of capturing the overall permeability trend (Fig. 9). However, the relatively high RMSE value (339.16 mD) reflects significant deviations in absolute permeability, particularly in the mid-range values. The log-scale RMSE of 0.63 further suggests that errors are more pronounced in certain permeability regimes. Visual inspection of the plot reveals clustering effects, where predictions tend to align along discrete bands corresponding to specific HFU classes, rather than forming a continuous distribution. This behavior highlights a key limitation of the HFU method, as it relies on discrete rock typing and fixed porosity–permeability relationships, leading to reduced sensitivity in transitional zones and underestimation or overestimation in heterogeneous intervals. Despite its simplicity and reasonable overall correlation, the HFU approach lacks the flexibility required to accurately model continuous variability in permeability.

**Physics Guided Neural Network Permeability Prediction**.

**Fig. 10:**
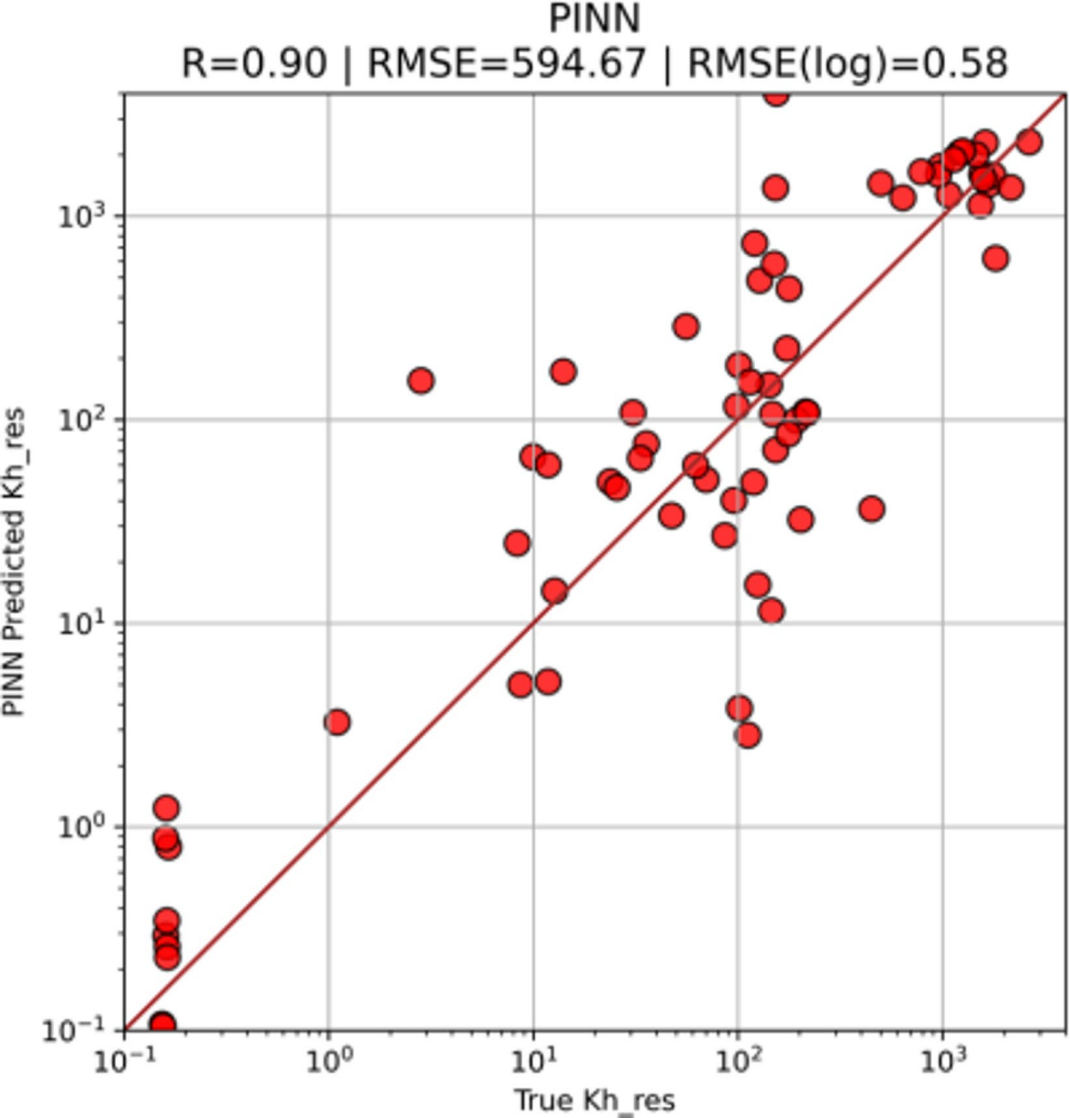
Crossplot of measured core permeability versus permeability predicted using the Physics Informed Neural Network model. The brown line represents the 1:1 reference line.

The crossplot of measured versus PINN-predicted permeability shows a strong overall correlation of $\:R\:=\:0.90$ (Fig. 10), indicating that the model successfully captures the general relationship between input features and permeability. Compared to the HFU method, the PINN produces a more continuous distribution of predictions, reflecting its ability to learn nonlinear patterns directly from the data while being guided by physical constraints. However, the relatively high RMSE value (594.67 mD) suggests significant deviations in absolute permeability, particularly at higher permeability ranges where overestimation is evident. The log-scale RMSE (0.58) indicates improved performance in capturing relative trends across orders of magnitude compared to the HFU method. Despite the incorporation of physics-based constraints during training, the model exhibits noticeable scatter, especially in low to intermediate permeability regions, highlighting limitations in fully enforcing physical consistency during inference. Overall, the PINN model provides a more flexible and continuous representation than the HFU approach, but still faces challenges in accurately predicting extreme permeability values.

**Mult-Tasking Physics Guided Neural Network Permeability Prediction**.

**Fig. 11:**
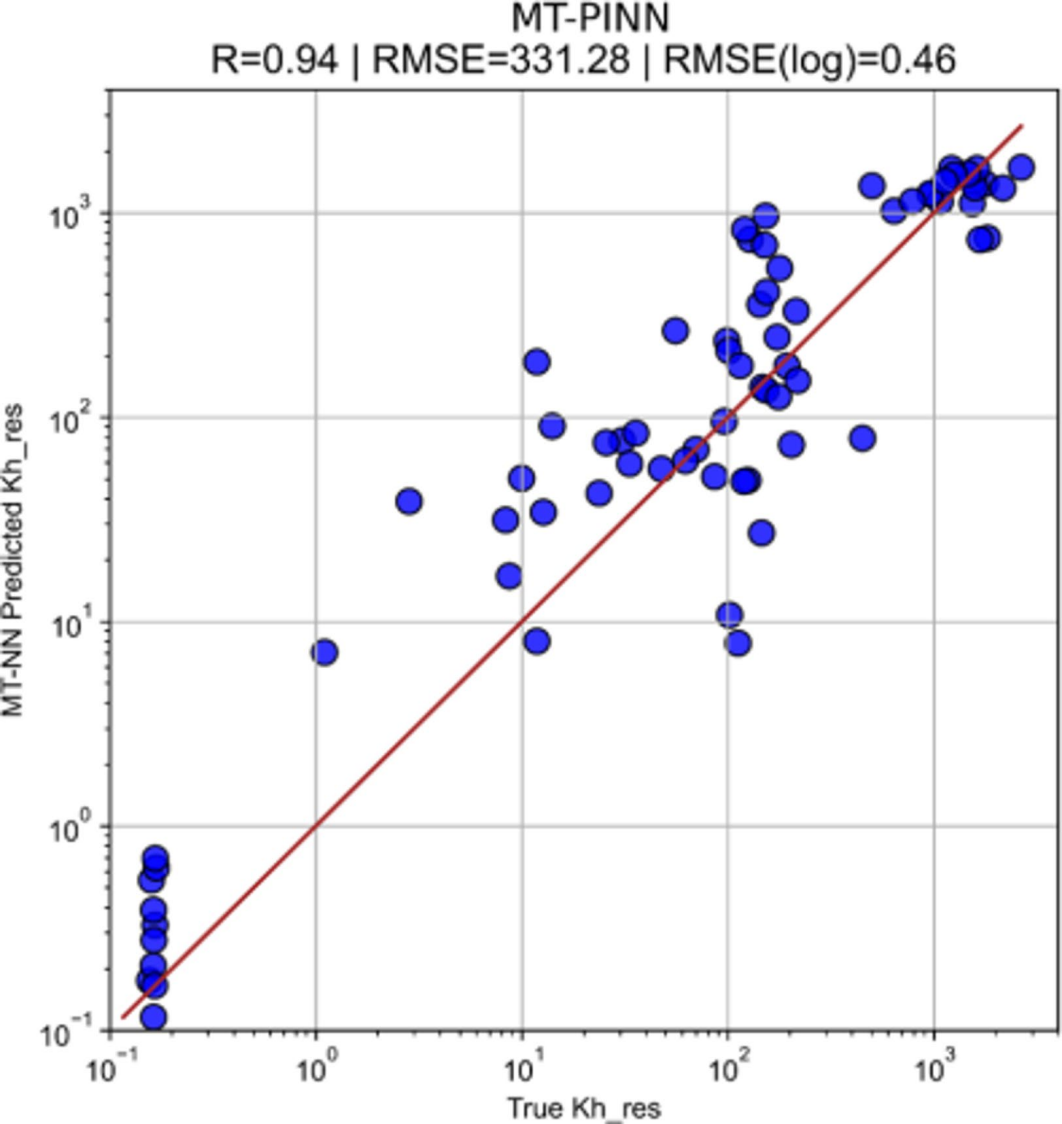
Crossplot of measured core permeability versus permeability predicted using the Multi-Tasking Physics Informed Neural Network model. The brown line represents the 1:1 reference line.

The MT-PINN model exhibits the best overall performance among the evaluated approaches, as evidenced by the crossplot of measured versus predicted permeability and a strong correlation coefficient of $\:R\:=\:0.936$ (Fig. 11). The model achieves a significantly improved balance between accuracy and stability, as reflected by a lower RMSE (331.28 mD) compared to the standard PINN and a reduced log-scale RMSE (0.46), indicating better consistency across different permeability magnitudes. Unlike the HFU method, which produces discrete clustering, and the standard PINN, which exhibits considerable scatter, the MT-PINN predictions show a more continuous and well-aligned distribution along the 45-degree line. This improvement is attributed to the integration of multi-task learning, where rock type prediction and permeability estimation are jointly optimized, along with the incorporation of physics-based constraints during both training and inference through probabilistic rock typing. The model demonstrates enhanced capability in capturing both low and high permeability regimes, with reduced bias and improved representation of transitional zones. Overall, the MT-PINN demonstrates improved permeability prediction consistency within the studied reservoir and partially addresses several limitations observed in both HFU and conventional PINN approaches.

To evaluate the sensitivity of the MT-PINN framework to loss-function weighting, multiple combinations of the regression, classification, and physics-guided loss coefficients were investigated. Model performance was evaluated on the blind test well using the correlation coefficient (R) and RMSE(log). The results demonstrated that the predictive performance of the framework was sensitive to the relative contribution of the three loss components, particularly the balance between permeability regression, probabilistic HFU classification, and the physics-guided petrophysical constraints.

The sensitivity analysis revealed that several weighting combinations produced consistently strong predictive performance, indicating stable optimization behavior across a range of loss-weight configurations. Among the evaluated cases, the configuration corresponding to.

= 0.3, $\:{\boldsymbol{\lambda\:}}_{\boldsymbol{R}\boldsymbol{T}}$ = 0.4, and $\:{\boldsymbol{\lambda\:}}_{\boldsymbol{p}\boldsymbol{h}\boldsymbol{y}\boldsymbol{s}}$ = 0.2 achieved the highest correlation coefficient (*R* = 0.936) while maintaining one of the lowest RMSE(log) values (0.462). Similarly, additional configurations with stronger classification weighting also produced comparable results, suggesting that the proposed MT-PINN framework remained robust under moderate variations in the loss balancing strategy.

Overall, the results indicate that balanced integration of data-driven learning, probabilistic HFU classification, and physics-guided constraints contributed to the most stable and accurate permeability predictions within the studied dataset. The top-performing configurations obtained from the sensitivity analysis are summarized in Table 7.

**Table 7:** Sensitivity analysis of MT-PINN loss-function weights.

$\:{\boldsymbol{\lambda\:}}_{\boldsymbol{d}\boldsymbol{a}\boldsymbol{t}\boldsymbol{a}}$	$\:{\boldsymbol{\lambda\:}}_{\boldsymbol{R}\boldsymbol{T}}$	$\:{\boldsymbol{\lambda\:}}_{\boldsymbol{p}\boldsymbol{h}\boldsymbol{y}\boldsymbol{s}}$	*R*	RMSE(log)
0.3	0.4	0.2	0.9367	0.462
0.4	1.0	0.5	0.9365	0.468
0.8	1.0	0.5	0.9364	0.461
0.1	0.7	0.4	0.9358	0.484
0.1	0.9	0.5	0.9355	0.489

**Fig. 12:**
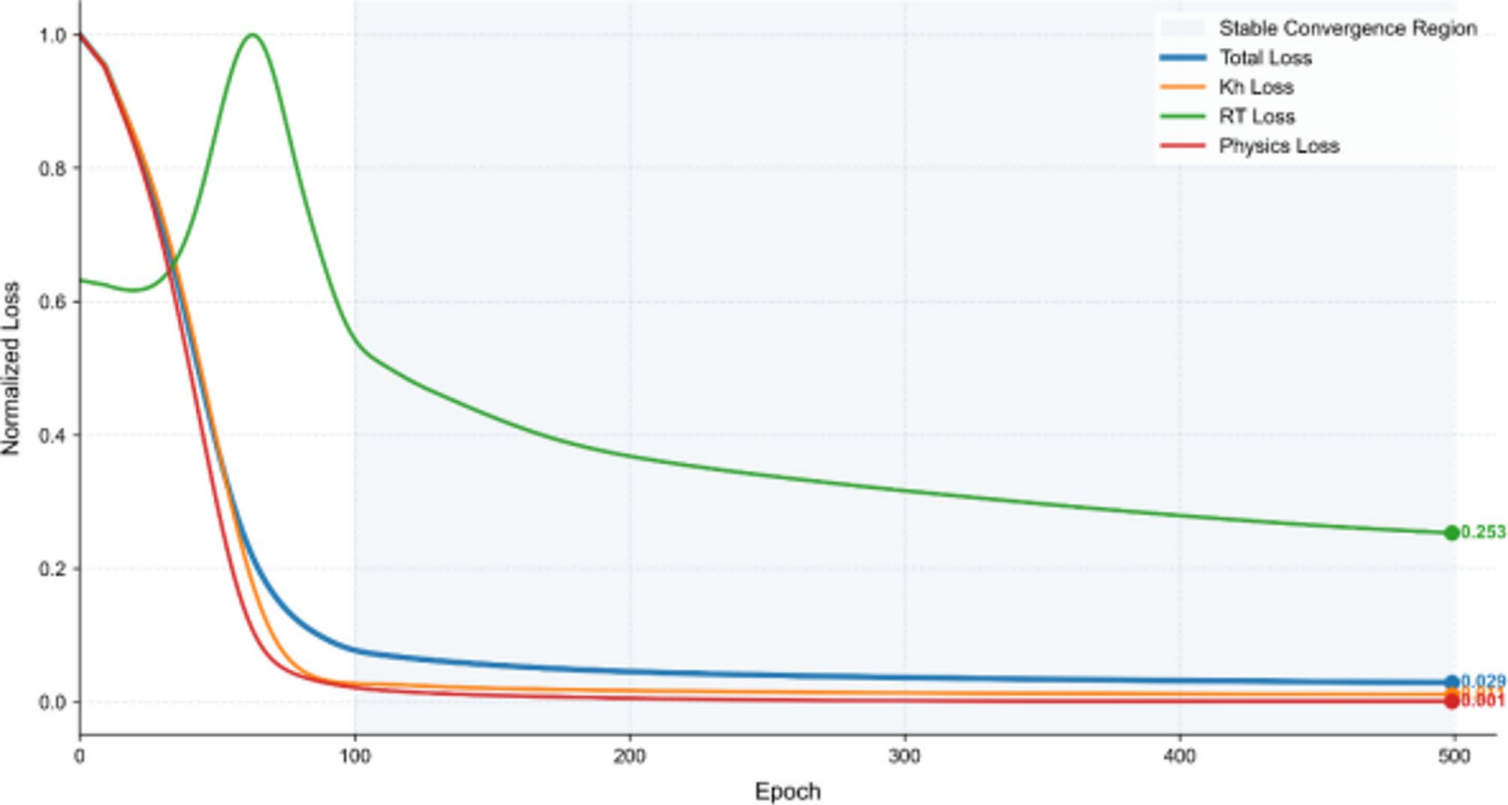
Training convergence behavior of the MT-PINN framework showing the evolution of normalized total loss, permeability prediction loss, HFU classification loss, and physics-guided loss during optimization. The highlighted region indicates the late-stage stable convergence interval observed after approximately 100 training epochs, demonstrating smooth and numerically stable multi-task optimization behavior.

The training convergence behavior of the MT-PINN framework is presented in Fig. 12. The normalized total loss, permeability prediction loss, HFU classification loss, and physics-guided loss exhibited stable and monotonic convergence throughout the optimization process without significant oscillation or divergence. Rapid loss reduction was observed during the early training stages, followed by gradual late-stage refinement. The physics-guided loss converged rapidly to a very low normalized value, indicating effective enforcement of the petrophysical consistency constraints during training.

A relatively stable convergence region was observed after approximately 100 training epochs, where all loss components demonstrated smooth and numerically stable optimization behavior. Although convergence stabilization occurred during the intermediate training stages, continued optimization up to 500 epochs provided additional refinement, particularly for complex and high-permeability samples. The HFU classification loss exhibited slower convergence compared to the permeability and physics-guided losses, reflecting the greater complexity of probabilistic rock-type discrimination within heterogeneous carbonate reservoirs.

No explicit regularization techniques such as dropout, weight decay, or early stopping were applied in the present study due to the intentionally compact network architecture and relatively limited number of trainable parameters (~ 5,000). Instead, model stability was supported through the integration of probabilistic HFU representations and physics-guided petrophysical constraints within the multi-task learning framework.

**Fig. 13:**
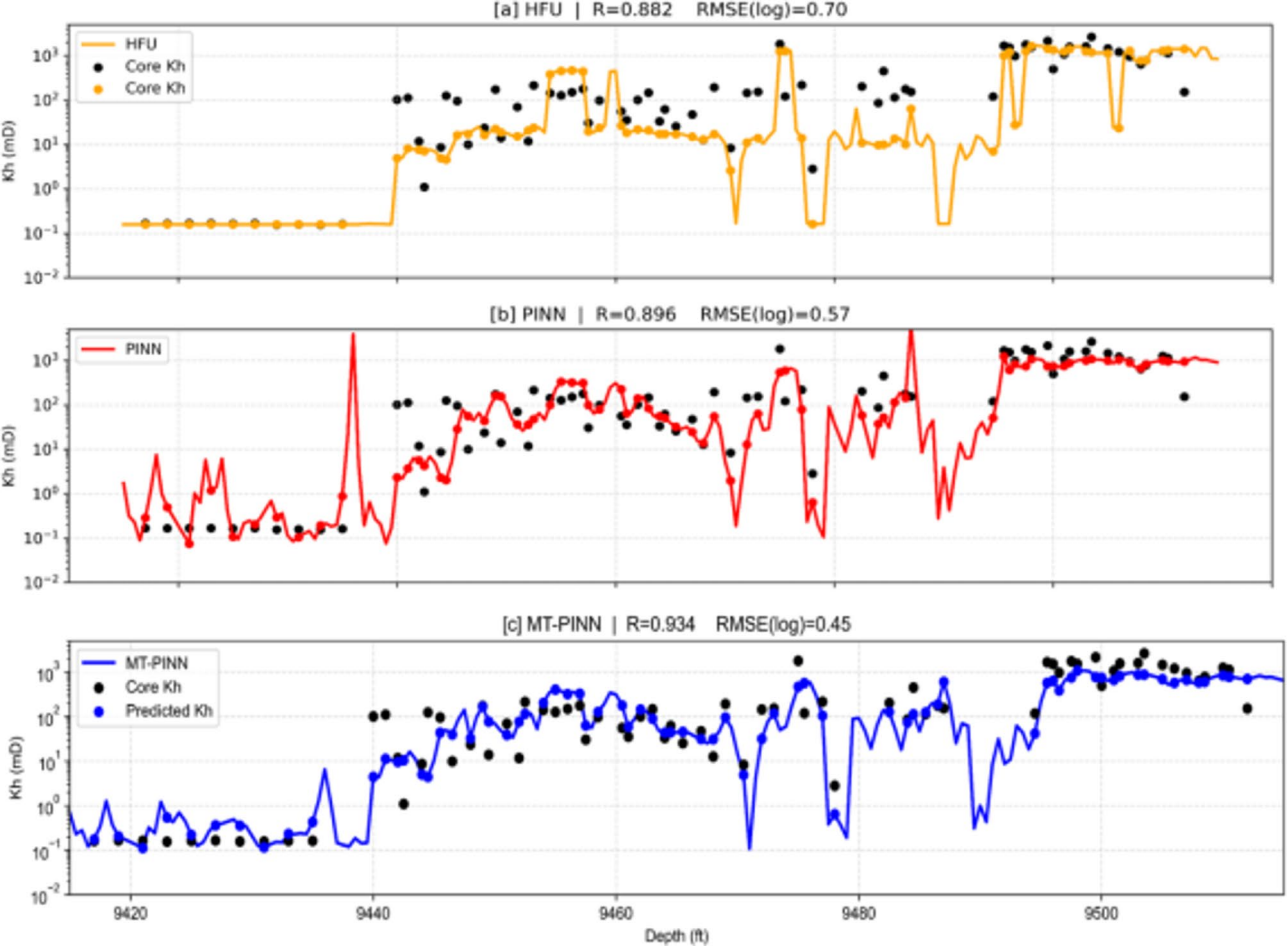
Depth comparison between measured core permeability and predicted permeability for Well Shahd SE-32. [a] Permeability predicted using the conventional HFU method, [b] Permeability predicted using PINN and [c] Permeability predicted using MT-PINN.

Figure 13 presents the permeability prediction results along depth for the blind testing well Shahd SE-32, comparing the HFU, PINN, and MT-PINN approaches against core measurements. The HFU method exhibits step-like behavior with abrupt transitions, reflecting its reliance on discrete rock typing and resulting in limited resolution in heterogeneous intervals. The standard PINN model provides a more continuous prediction; however, it shows noticeable fluctuations and instability in certain depth intervals, particularly in low to intermediate permeability zones. In contrast, the MT-PINN model demonstrates the most consistent and reliable performance, closely following the core measurements across the entire depth range. It effectively captures both large-scale trends and local variations, with reduced noise and improved continuity. This behavior highlights the advantage of integrating probabilistic rock typing and physics-guided learning within a multi-task framework, enabling a more accurate and geologically realistic representation of permeability variations along the well.

**Fig. 14:**
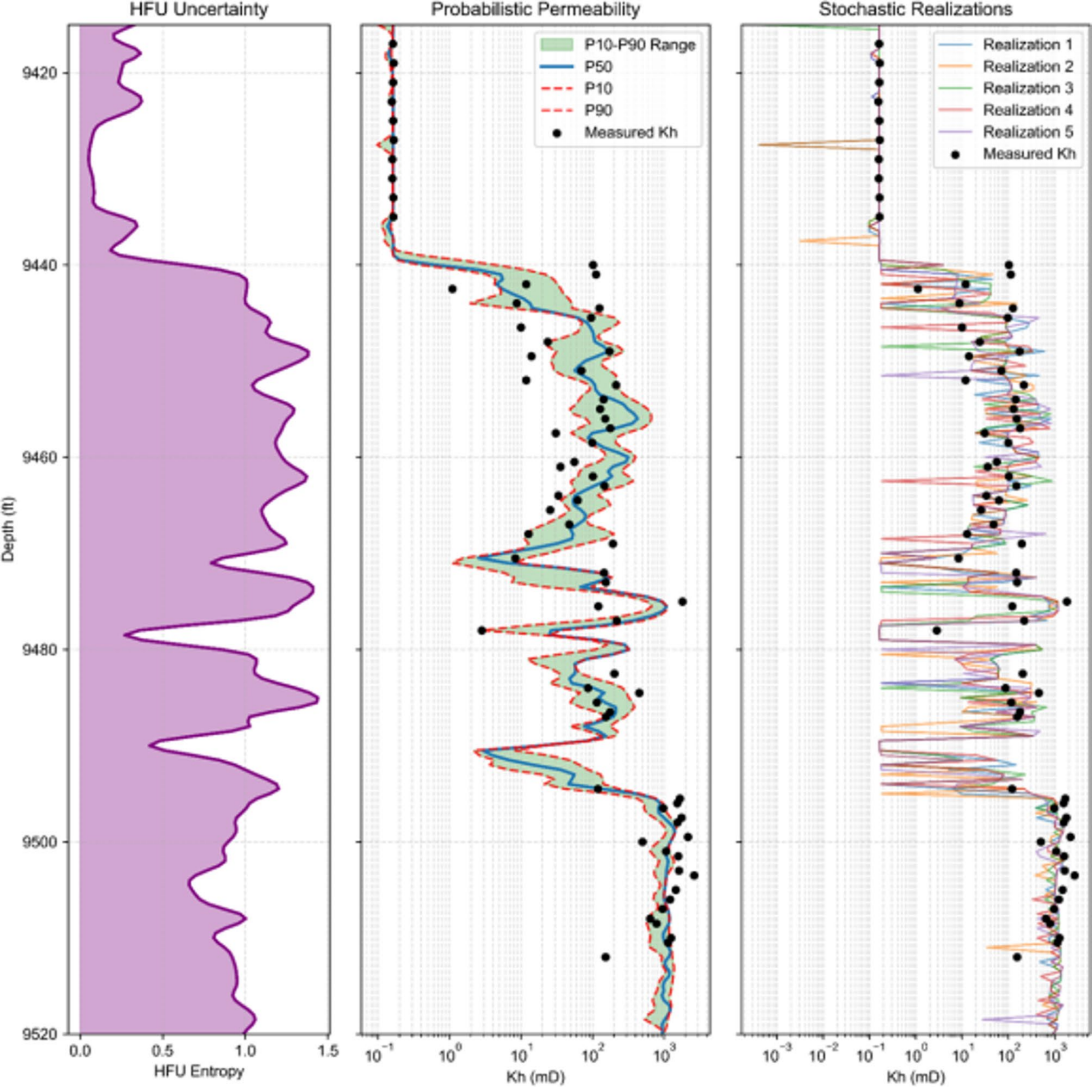
Entropy-based HFU uncertainty, probabilistic permeability distribution, and stochastic permeability realizations generated for the blind test well Shahd SE-32 using the MT-PINN framework. The central panel shows the P10–P90 permeability envelope and P50 realization derived from stochastic sampling of probabilistic HFU predictions, while the right panel presents multiple stochastic permeability realizations. Increased uncertainty and realization variability are observed within heterogeneous and transitional reservoir intervals characterized by elevated entropy values.

To further evaluate the uncertainty associated with probabilistic HFU predictions, entropy-based uncertainty analysis and stochastic permeability realizations were generated for the blind test well Shahd SE-32 (Fig. 14). The entropy profile reveals increased uncertainty within heterogeneous and transitional reservoir intervals characterized by overlapping petrophysical responses and mixed HFU behavior. These intervals correspond to wider P10–P90 permeability envelopes and increased variability among stochastic permeability realizations. In contrast, lower entropy intervals exhibit narrower uncertainty ranges and more stable permeability predictions. The probabilistic realizations successfully capture much of the variability observed in the measured permeability data, demonstrating the capability of the MT-PINN framework to propagate HFU uncertainty into permeability prediction. This probabilistic representation provides additional flexibility compared to deterministic HFU assignment and highlights the potential applicability of the proposed framework for uncertainty-aware reservoir characterization and multi-realization reservoir modeling workflows.

**Ablation Study**.

To evaluate the contribution of the individual components of the proposed framework and compare its performance against a conventional machine learning baseline, a comparative ablation study was performed using multiple model configurations. The evaluated models included: (1) a Random Forest (RF) regression model representing a conventional nonlinear machine learning baseline, (2) a standard multilayer perceptron (MLP), (3) a PINN model incorporating physics-guided petrophysical constraints, (4) a multi-task neural network (MT-NN) combining permeability prediction and HFU classification without physics constraints, and (5) the full MT-PINN framework. The corresponding results are summarized in Table 8.

The Random Forest model achieved moderate predictive performance (*R* = 0.847; RMSE(log) = 0.699), demonstrating the capability of conventional machine learning approaches for nonlinear permeability prediction under limited-data conditions. Similarly, the MLP model produced weaker overall performance (*R* = 0.808; RMSE(log) = 0.810), highlighting the limitations of purely data-driven neural network approaches when applied to sparse and heterogeneous subsurface datasets.

Introducing physics-guided constraints through the PINN configuration significantly improved prediction accuracy and stability relative to both RF and MLP models, reducing the logarithmic prediction error to 0.60 and increasing the correlation coefficient to 0.88. The MT-NN model, which incorporated multi-task learning and HFU classification without physics constraints, achieved intermediate performance (*R* = 0.872; RMSE(log) = 0.642. Although MT-NN improved performance relative to purely data-driven baselines, its predictive stability remained inferior to the full MT-PINN framework.

The full MT-PINN framework achieved the best overall predictive performance, with the highest correlation coefficient (*R* = 0.936) and the lowest RMSE(log) value (0.462). These results demonstrate that the combined integration of probabilistic HFU representations, multi-task optimization, and physics-guided petrophysical constraints contributed to the most stable and accurate permeability predictions within the studied dataset.

**Table 8:** Ablation study of MT-PINN components.

Metric	RF	MLP	PINN	MT-NN	MT-PINN
Physics Constraint	✗	✗	✓	✗	✓
HFU Classification	✗	✗	✗	✓	✓
Probabilistic HFU	✗	✗	✗	✗	✓
Multi-task Learning	✗	✗	✗	✓	✓
Model Type	Conventional ML	Neural Network	PINN	Multi-task NN	Proposed Framework
R	0.847	0.808	0.88	0.872	0.936
RMSE(log)	0.699	0.810	0.60	0.642	0.462

**Leave-One-Well-Out Validation**

To further evaluate the cross-well consistency of the proposed framework, a leave-one-well-out (LOO) validation scheme was performed, in which each well was used as a blind test well while the remaining wells were used for training. The corresponding results are summarized in Table 9.

The MT-PINN model demonstrated relatively consistent predictive performance across the three leave-one-well-out (LOO) validation scenarios, with correlation coefficients ranging from 0.912 to 0.939 and RMSE(log) values between 0.366 and 0.502. The overall average performance across the three blind-test wells yielded a mean correlation coefficient of 0.929 ± 0.015 and a mean RMSE(log) of 0.447 ± 0.071, indicating stable predictive behavior and limited performance variability between wells.

Among the evaluated wells, Shahd SE-32 was selected as the primary blind test well presented in the main workflow because it contains a broader distribution of low-, medium-, and high-permeability intervals while also representing the smallest portion of the available dataset (~ 23%). This configuration provides a more challenging validation scenario under limited-data conditions.

Although the available dataset remains limited, the LOO validation results provide additional evidence supporting the robustness, transferability, and generalization capability of the proposed MT-PINN framework within the studied heterogeneous carbonate reservoir.

**Table 9:** Leave-one-well-out validation results for the MT-PINN model.

Blind Test Well	*R*	RMSE	RMSE(log)
Shahd SE-32	0.936	331.28	0.462
Shahd SE-42	0.912	432.2	0.502
Shahd SE-05	0.939	411.1	0.366

**Repeated-Run Robustness Evaluation**

To further evaluate the numerical stability and reproducibility of the proposed MT-PINN framework, repeated training runs were performed using five different random initialization seeds. The corresponding results are summarized in Table 10. The repeated runs demonstrated relatively limited variability in predictive performance, with correlation coefficient values ranging from 0.901 to 0.936 and RMSE(log) values ranging from 0.462 to 0.601.

The overall average performance across all realizations yielded a mean correlation coefficient of 0.912 ± 0.015, a mean RMSE of 430.27 ± 105.6 mD, and a mean RMSE(log) of 0.550 ± 0.056, indicating stable optimization behavior and consistent permeability prediction despite variations in network initialization. The best-performing realization corresponded to Seed 123, which achieved the highest correlation coefficient (*R* = 0.936) and the lowest RMSE(log) value (0.462).

Overall, the relatively small variation observed between repeated runs suggests that the predictive improvements achieved by the MT-PINN framework are not solely dependent on a specific random initialization, supporting the robustness and reproducibility of the proposed workflow.

**Table 10:** Repeated-run robustness evaluation of MT-PINN.

Seed	*R*	RMSE (mD)	RMSE(log)
42	0.903	412.99	0.576
7	0.901	607.81	0.585
21	0.919	423.37	0.528
100	0.901	375.91	0.6012
123	0.936	331.28	0.462
Mean ± Std	0.912 ± 0.015	430.27 ± 105.6	0.550 ± 0.056

## Discussion

The results highlight distinct differences between the HFU, PINN, and MT-PINN approaches in modeling permeability. The HFU method, while physically grounded, is limited by its reliance on discrete rock typing and fixed porosity–permeability relationships, leading to step-like predictions and reduced sensitivity to heterogeneity. The standard PINN improves prediction continuity by incorporating data-driven learning with petrophysical consistency constraints; however, these constraints are enforced only during the training stage through the loss function and are not explicitly imposed during prediction. Consequently, the model still exhibits noticeable scatter and instability, particularly within low- and intermediate-permeability regions.

The MT-PINN model overcomes these limitations by integrating multi-task learning with probabilistic rock typing and physics-guided constraints. By jointly predicting permeability and HFU classes, along with their associated probabilities, the model enables both deterministic and probabilistic permeability estimation, reducing sensitivity to classification errors and providing smoother transitions across flow units. This results in improved accuracy and a more realistic representation of reservoir heterogeneity, as demonstrated in both crossplot and depth-based evaluations.

A key advantage of the MT-PINN framework is its ability to generate probabilistic HFU outputs, which can be directly incorporated into 3D stochastic reservoir modeling for multi-realization analysis and uncertainty quantification. Despite these improvements, challenges remain in accurately capturing very low-permeability regimes, highlighting opportunities for future enhancements. Overall, the MT-PINN approach demonstrates the potential to integrate traditional HFU methods with data-driven modeling within the studied reservoir. Although the current study is limited by dataset size, the results indicate that the proposed framework may provide a promising workflow for permeability prediction and uncertainty-aware reservoir characterization.

The novelty of the proposed framework primarily lies in the unified integration of probabilistic HFU learning, multi-task permeability prediction, and physics-guided petrophysical constraints within a single reservoir characterization workflow.

It should be noted that the present work is based on a limited number of wells and should therefore be interpreted as a case-study investigation rather than a universally validated framework. Additional validation using larger and more diverse datasets is recommended in future studies.

## Conclusions

This study demonstrates the effectiveness of a physics-informed multi-task neural network (MT-PINN Algorithm) for permeability prediction, combining data-driven learning with physical constraints and rock typing within a unified framework. Compared to conventional HFU and standard PINN approaches, the proposed method provides improved consistency and predictive capability across different permeability regimes.

The key advantages of the MT-PINN model can be summarized as follows:

- Simultaneous prediction of permeability and hydraulic flow units (HFUs) within a single framework.
- Integration of probabilistic rock typing, enabling smooth transitions between flow units.
- Improved prediction accuracy and reduced error compared to HFU and standard PINN models.
- Enhanced representation of reservoir heterogeneity through continuous predictions.
- Generation of class probabilities that can be directly used in 3D stochastic modeling and multi-realization workflows.

These features demonstrate the potential of the MT-PINN algorithm as a promising approach for permeability prediction and uncertainty-aware reservoir characterization within the studied reservoir.

## Data Availability

The raw data supporting the results of this study was obtained from the Egyptian General Petroleum Corporation. Data are, however, available from the corresponding author upon reasonable request and with permission of the Egyptian General Petroleum Corporation.

## Declarations

### Walid M. Mabrouk

Supervision, Introduction, Results & Discussion, Conclusion.

### Ahmed M. Metwally

Introduction, Methodology, Model Training and Evaluation, Results & Discussion, Conclusion.
